# The effects of age and systemic metabolism on anti-tumor T cell responses

**DOI:** 10.7554/eLife.62420

**Published:** 2020-11-10

**Authors:** Jefte M Drijvers, Arlene H Sharpe, Marcia C Haigis

**Affiliations:** 1 Department of Immunology, Blavatnik Institute and Ludwig Center at Harvard, Harvard Medical SchoolBostonUnited States; 2 Evergrande Center for Immunologic Diseases, Harvard Medical School and Brigham and Women’s HospitalBostonUnited States; 3 Department of Cell Biology, Blavatnik Institute and Ludwig Center at Harvard, Harvard Medical SchoolBostonUnited States; University of WashingtonUnited States; Weill Cornell MedicineUnited States

**Keywords:** immunity, metabolism, cancer metabolism, obesity, aging

## Abstract

Average age and obesity prevalence are increasing globally. Both aging and obesity are characterized by profound systemic metabolic and immunologic changes and are cancer risk factors. The mechanisms linking age and body weight to cancer are incompletely understood, but recent studies have provided evidence that the anti-tumor immune response is reduced in both conditions, while responsiveness to immune checkpoint blockade, a form of cancer immunotherapy, is paradoxically intact. Dietary restriction, which promotes health and lifespan, may enhance cancer immunity. These findings illustrate that the systemic context can impact anti-tumor immunity and immunotherapy responsiveness. Here, we review the current knowledge of how age and systemic metabolic state affect the anti-tumor immune response, with an emphasis on CD8^+^ T cells, which are key players in anti-tumor immunity. A better understanding of the underlying mechanisms may lead to novel therapies enhancing anti-tumor immunity in the context of aging or metabolic dysfunction.

## Introduction

Average human lifespan has dramatically increased across the globe. For example, life expectancy in the United States (U.S.) has risen by more than 30 years since the beginning of the 20^th^ century, from 47 to 79 years (Arias and Xu, 2019). More recently, many parts of the world have seen a sharp increase in obesity rates, with now over 40% of adults in the U.S. being obese (defined as a body mass index (BMI) ≥30 kg/m^2^). These profound demographic changes are also reflected in the diseases that impose the largest burden on present-day societies. Notably, cancer is the leading cause of death in the U.S. for individuals between 55 and 74 years of age and the second leading cause of death overall, behind only cardiovascular disease (Kochanek et al., 2019).

Age is among the most well-known cancer risk factors, and the incidence of most cancer types increases with age (Siegel et al., 2019). Illustratively, the chance for an individual in the U.S. to develop any cancer in the first 50 years of life is 4–5%, whereas this is close to 30% over the age of 70 (Siegel et al., 2019). Similarly, obesity is a risk factor for at least 13 cancer types, including common cancers like colorectal and postmenopausal breast cancer (Lauby-Secretan et al., 2016). Due to the increasingly high prevalence of obesity in the western world, obesity-related cancers constitute a significant health problem: between 2011 and 2015, almost 5% and almost 10% of cancer cases in men and women respectively of 30 years and older in the U.S. were attributable to excess body weight (Islami et al., 2018). In contrast to aging and obesity, dietary restriction is thought to provide widespread health benefits and increased lifespan (Fontana and Partridge, 2015; López-Otín et al., 2016; McCay et al., 1935), and the limited evidence available, largely derived from animal studies, suggests that cancer incidence may also be lowered with dietary restriction (Colman et al., 2009; Michels and Ekbom, 2004; Ross and Bras, 1965; Rous, 1914; Weindruch and Walford, 1982).

For both aging and obesity, a plethora of systemic and local factors have been suggested to directly promote cancer incidence and growth (Fane and Weeraratna, 2020; Khandekar et al., 2011). Although aging and excess body weight are distinct physiological entities, both conditions are also associated with reduced immune function, as evidenced by increased susceptibility to infections and suboptimal antibody titers following vaccination (Nikolich-Žugich et al., 2012; Painter et al., 2015; Sheridan et al., 2012), and both are among the main risk factors for a severe disease course in the ongoing COVID-19 pandemic, which is characterized by immune dysregulation and dysfunction (Lighter et al., 2020; Vabret et al., 2020; Wu and McGoogan, 2020). Given the recent appreciation of the importance of anti-cancer immune responses, this poses the question whether altered immunity may impact cancer rates and outcomes in systemic conditions like aging, obesity, and dietary restriction.

The first evidence for cancer immunotherapy was provided by the American surgeon William Coley in the late 19^th^ century (McCarthy, 2006). Dr. Coley injected sarcoma patients with streptococcal bacteria or bacterial products, inducing an immune response and, in some cases, tumor regressions. Not much later, the notion that defense mechanisms must exist in the body to limit the frequency of malignancies was first suggested by Ehrlich, 1909. However, Coley’s approach fell out of favor, and it was not until the second half of the 20^th^ century that experimentation with immune-mediated cancer therapies, including the first cancer vaccines, resumed (Decker et al., 2017; Graham and Graham, 1959). Around the same time, the concept of cancer immunosurveillance was described by Burnet and Thomas but could not yet be experimentally proven given the limited understanding of the immune system and the tools available at the time (Burnet, 1957; Dunn et al., 2002). The field of immunology was then propelled by several seminal discoveries, including the existence of distinct T and B lymphocyte populations (Cooper et al., 1965; Miller et al., 1967) as well as the concept of major histocompatibility complex (MHC) restriction in T cell-mediated immunity (Zinkernagel and Doherty, 1974). Definitive evidence for a role of immune cells in suppressing malignant tumors was provided by the laboratory of Robert Schreiber, who observed that tumors generated in immunodeficient mice could be cleared upon transplantation into immunocompetent animals (Shankaran et al., 2001), confirming the existence of cancer immunosurveillance and immunoediting (Dunn et al., 2002). These findings led to a renewed interest and confidence in the field of cancer immunotherapy, which would ultimately revolutionize cancer care (see ‘Cancer immunotherapies’ section below).

CD8^+^ T cells play a central role in anti-tumor immune responses by recognizing tumor cell antigens that differ from normal tissue and deploying cytolytic machinery to kill cancer cells directly. However, CD8^+^ T cell functions are often suppressed in the context of anti-tumor immunity. One reason for reduced T cell function in this context is the potently immunosuppressive nature of the tumor microenvironment (TME), where inhibitory signaling pathways are activated and immunosuppressive cell populations are present (Nakamura and Smyth, 2020). Moreover, extensive research has highlighted the importance of cell-intrinsic metabolic pathways for T cell activation, differentiation, and function (Buck et al., 2017). Since the TME is often depleted of nutrients, due to limited blood supply and competition with metabolically active tumor cells, nutrient unavailability provides another layer of immunosuppression (Lim et al., 2020).

In addition to local factors, the macroenvironment in which anti-tumor T cell responses take place also warrants consideration. Interestingly, aging and obesity both induce metabolic (e.g. insulin resistance) and immunologic (e.g. chronic inflammation) systemic alterations (Hotamisligil, 2017; López-Otín et al., 2013). However, how these organism-level changes are reflected in the TME and impact the anti-tumor immune response both systemically and locally are incompletely understood. Given that average age and obesity prevalence are rising in many parts of the world, a better understanding of these mechanisms is important to inform therapeutic strategies aiming to enhance the anti-tumor immune response in patients of any age and body composition. Here, we review the current knowledge of the shared and distinct effects of age, obesity, and various forms of dietary restriction on anti-tumor T cell responses with an emphasis on the metabolic pathways involved and the CD8^+^ T cell compartment. For each of these conditions, we will address alterations in (1) the anti-tumor T cell response, (2) metabolism and signaling, (3) TME-specific immunosuppression, and (4) cancer immunotherapy responsiveness.

### Anti-tumor T cell responses

T lymphocytes are a part of the adaptive immune system, which is characterized by the ability to mount immune responses that are highly specific to certain antigens and result in the formation of immunological memory (Chaplin, 2010). To facilitate the recognition of any pathogenic antigen, T cells are endowed with unique antigen receptors known as T cell receptors (TCRs). Since TCRs recognize antigenic peptides in the context of MHC molecules, they rely on other cells to take up, process, and present antigens to them. To facilitate the interaction of the TCR with peptide-MHC complexes, T cells express a coreceptor, CD8 or CD4, which binds to MHC class I (MHC-I) or MHC class II (MHC-II) respectively (Rossjohn et al., 2015).

**Figure 1.**
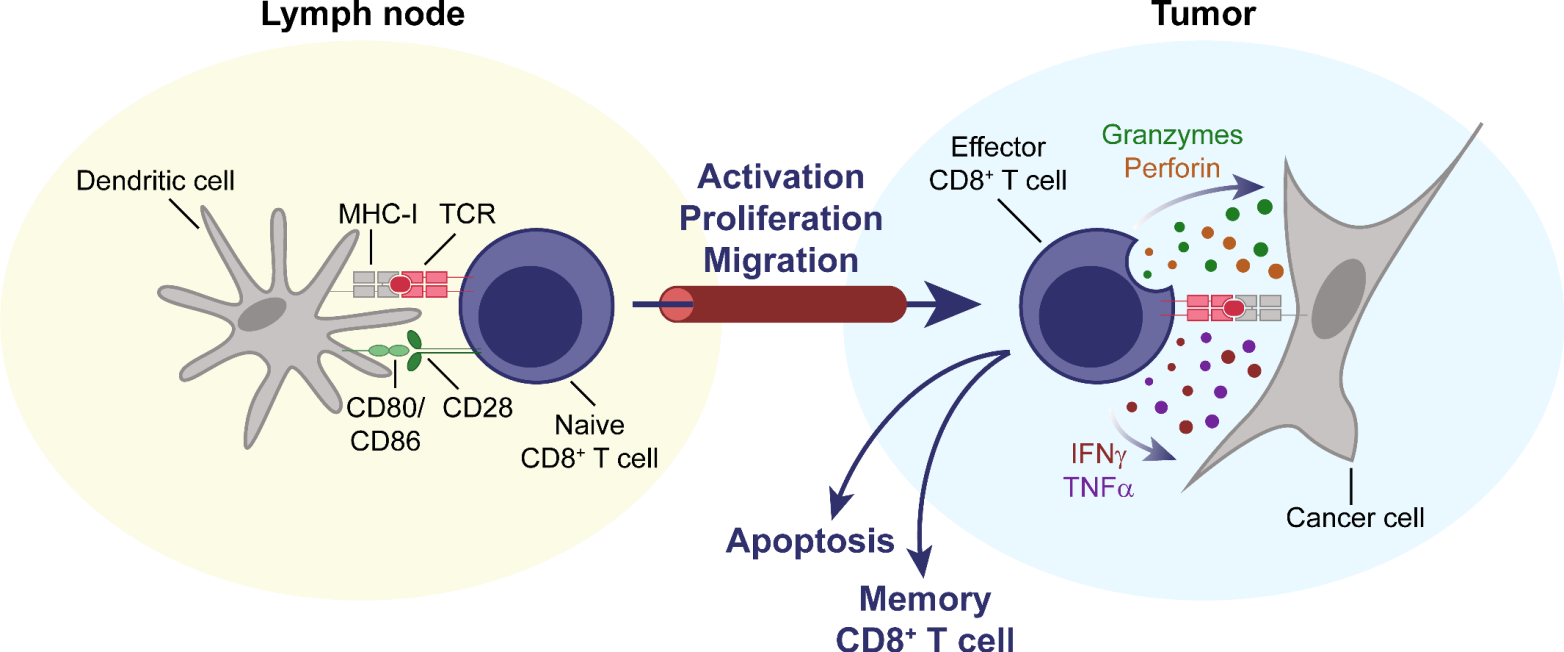
CD8^+^ T cells are key effectors of the anti-tumor immune response. Naïve CD8^+^ T cells circulate through the body, until their cognate antigen is presented to them by an antigen-presenting cell, for example a dendritic cell, in a secondary lymphoid organ, for example a lymph node. T cells then become activated, proliferate, differentiate, and migrate to the tumor. Upon entering the TME, they can mediate an effective anti-tumor response through direct cytolytic activity, mediated by perforin and granzymes, and the secretion of cytokines like IFNγ and TNFα. IFNγ, interferon γ. MHC-I, major histocompatibility complex I. TCR, T cell receptor. TME, tumor microenvironment. TNFα, tumor necrosis factor α.

Naïve CD8^+^ and CD4^+^ T cells are generated from bone marrow-derived progenitor cells in the thymus and circulate through secondary lymphoid organs in the body, such as lymph nodes and spleen, until they encounter their cognate antigen. For initial T cell activation, an antigen needs to be presented by a professional antigen-presenting cell (APC), usually a cell of the myeloid lineage like a dendritic cell or macrophage, in the context of the appropriate costimulatory signals, most importantly ligation of the costimulatory receptor CD28 (Esensten et al., 2016; Pennock et al., 2013). Upon activation, a T cell undergoes clonal expansion, differentiates into an effector cell, and migrates toward the relevant peripheral tissue, for example a site of infection or a tumor (Smith-Garvin et al., 2009). Cytotoxic effector CD8^+^ T cells can directly kill cells that present their cognate antigen through cytotoxic molecules, like granzyme B (GzmB) and perforin, and also secrete pro-inflammatory cytokines, like interferon (IFN)-γ and tumor necrosis factor (TNF)-α (Farhood et al., 2019; Figure 1). CD8^+^ T cells therefore play a central role in the effectuation of immune responses against intracellular pathogens and cancer. Activated CD4^+^ T cells take on more diverse roles. Conventional CD4^+^ T cells (Tconvs) can differentiate into various types of immune helper T cells that support an immune response. A distinct lineage of CD4^+^ T cells, so-called regulatory T cells (Tregs), suppresses immune responses and is important for preventing autoimmunity (van der Vliet and Nieuwenhuis, 2007). After a successful immune response, most effector T cells undergo apoptosis, while some remain as memory T cells, which are poised to be reactivated more quickly than naïve T cells (Pennock et al., 2013).

In addition to the TCR and costimulatory receptors such as CD28, which promote T cell activation, T cells also express co-inhibitory receptors, including cytotoxic T lymphocyte-associated protein 4 (CTLA-4) and programmed death-1 (PD-1). Inhibitory signaling through these receptors is important to maintain tolerance to self-antigens and for the resolution of immune responses (Nishimura et al., 1999; Tivol et al., 1995). However, enhanced expression of co-inhibitory receptors develops when T cells are exposed to persistent antigen stimulation (e.g. during chronic viral infection or in tumors) and correlates with an exhausted, dysfunctional state in T cells (Barber et al., 2006; Blackburn et al., 2009). Moreover, tumor cells and other cell populations in the TME suppress T cell function by expressing ligands of these receptors, such as PD-L1 (Dong et al., 2002).

Several other immune cell types can also play important roles in anti-tumor immunity. Natural killer (NK) lymphocytes, like CD8^+^ T cells, can acquire a cytotoxic phenotype and kill cancer cells (Miller and Lanier, 2019). Some myeloid cells in the TME can promote the anti-tumor immune response, for example macrophages with a pro-inflammatory phenotype often referred to as ‘M1’, but many tumor-associated macrophages have an ‘M2’-like phenotype and have pro-tumor effects, including suppression of anti-tumor immunity (Zhou et al., 2020). Importantly, the M1 and M2 designations do not describe distinct populations, but rather reflect opposite ends of the wide spectrum of functional states that macrophages can assume (Murray, 2017). Additionally, myeloid-derived suppressor cells (MDSCs), a heterogenous population of immature myeloid cells, inhibit T cell function in the TME through a variety of mechanisms, including depletion of extracellular arginine, expression of PD-L1, and recruitment of Tregs (Kumar et al., 2016).

### Anti-tumor T cell responses with aging

Aging negatively impacts CD8^+^ T cell immunity, in cancer and other contexts, through a combination of T cell-intrinsic and -extrinsic mechanisms. First, the generation of new naïve T cells declines with age, as the thymus involutes and produces fewer naïve CD4^+^ and CD8^+^ T cells (Bains et al., 2009). Maintenance of the naïve T cell pool, both in terms of numbers and TCR diversity, thus becomes dependent on homeostatic proliferation of existing T cells, which leads to a reduction in naïve T cell numbers, especially of CD8^+^ T cells (Goronzy and Weyand, 2019). In addition, aging-associated adipocyte accumulation in the bone marrow also contributes to reduced hematopoiesis with age (Ambrosi et al., 2017), and hematopoiesis becomes skewed toward myeloid and away from lymphoid lineages with age (Beerman et al., 2010).

While the number of naïve T cells decreases with age, there are increased numbers of circulating T cells that express markers of antigen experience, for example CD45RO in humans or CD44 in mice (Lages et al., 2010; Li et al., 2019). Adoptive transfer experiments in mice have shown that these cells are dysfunctional (Decman et al., 2010). Some antigen-experienced cells express co-inhibitory receptors, including PD-1, and have been likened to exhausted T cells (Decman et al., 2012; Lages et al., 2010), whereas others are virtual memory T cells (Tvm), which develop from naïve T cells without full antigenic stimulation, and display some features of cellular senescence – an aging-associated phenotype of growth arrest (Chiu et al., 2013; Quinn et al., 2018; Renkema et al., 2014). However, although Tvm cells display a strongly reduced proliferation capacity, especially after TCR stimulation, they can still proliferate after stimulation with interleukin (IL)−15 (Quinn et al., 2018; Renkema et al., 2014), and thus do not have the irreversible cell cycle block that is characteristic of true cellular senescence (Goronzy and Weyand, 2019). In humans the number of T effector memory CD45RA (T_EMRA_) cells increases with age (Quinn et al., 2018). T_EMRA_ cells develop upon viral infections and display some, but not all, properties of cellular senescence (Goronzy and Weyand, 2019). Finally, a loss of the co-stimulatory receptor CD28, which is essential for normal T cell activation, has been observed on CD8^+^ T cells during aging (Lages et al., 2010), and CD8^+^ CD28^–^ T cells may even play an immunosuppressive role in tumors (Filaci et al., 2007).

**Figure 2.**
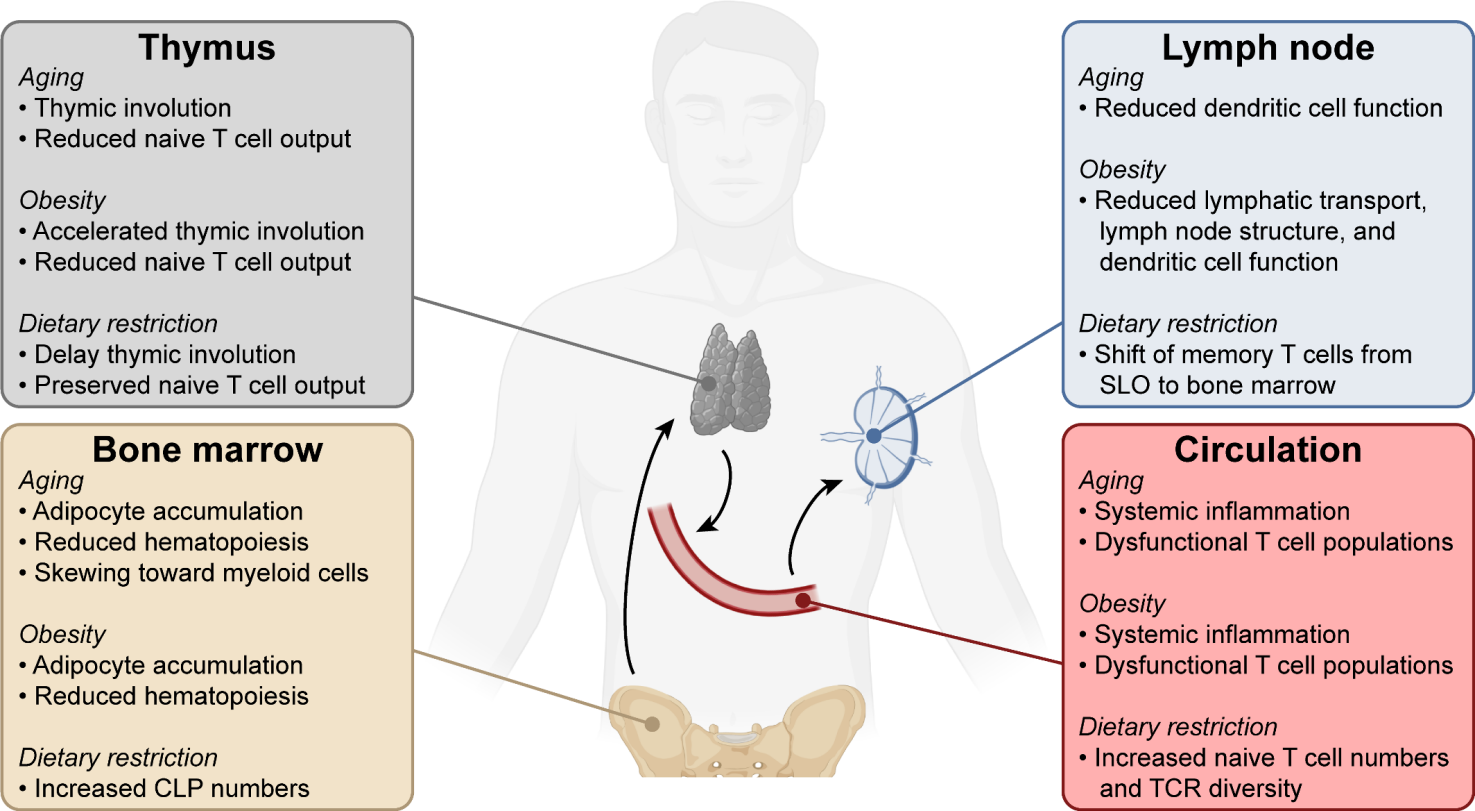
Systemic changes in the immune system with aging, obesity, and dietary restriction. T cell progenitors arise in the bone marrow and travel to the thymus where they develop into mature naïve T lymphocytes. Naïve T cells circulate through the bloodstream and secondary lymphoid organs. Systemic conditions like aging, obesity, and dietary restriction affect the immune system at each of these levels, impacting both anti-tumor and other immune responses. Aging- and obesity-associated changes lead to reduced T cell immunity, while dietary restriction-associated changes promote T cell responses. CLP, common lymphoid progenitor. SLO, secondary lymphoid organs. TCR, T cell receptor.

T cells rely on antigen-presenting cells, especially dendritic cells, to display antigens and provide costimulatory signals to drive T cell activation in secondary lymphoid organs. In addition to the described changes in the T cell compartment itself, dendritic cells also have reduced functionality with aging, including in the context of murine cancer models (Grolleau-Julius et al., 2008; Grolleau-Julius et al., 2006). Overall, a reduction in the naïve CD8^+^ T cell compartment, the existence of multiple dysfunctional T cell populations and reduced antigen presentation by dendritic cells likely all contribute to reduced T cell-mediated immunity in aging (Figure 2).

Few studies have analyzed the anti-tumor T cell response without any immune-boosting therapies in aged animals, but the existing studies predominantly show reduced T cell function in this context. For example, anti-tumor T cell responses are decreased with age in a mouse model of oral cancer, and this is accompanied by increased numbers of MDSCs (Sekido et al., 2019). Similarly, MDSCs inhibit anti-tumor T cell function with age in a murine breast cancer model (Grizzle et al., 2007). Defects in anti-tumor CD8^+^ T cell responses were also observed in a hematological cancer model as well as a mesothelioma model (Jackaman et al., 2019; Lustgarten et al., 2004). Paradoxically, one study found that the growth of several tumor types was slower in aged mice, and this difference in growth rate was CD8^+^ T cell-dependent (Oh et al., 2018). It is not clear what explains this discrepancy, but differences in experimental setup (e.g. mouse strain, tumor model, duration of aging) and environmental factors (e.g. microbiome) between studies could play a role. More research carefully examining the anti-tumor immune response in aged animals and humans is thus needed. At this point, most of the available evidence points toward a reduction of anti-tumor immunity with advanced age.

### Anti-tumor T cell responses with obesity

In addition to other systemic changes, including metabolic alterations and inflammation, obesity has been associated with reduced immune function (Figure 2). Some of the mechanisms impairing systemic CD8^+^ T cell function with age are promoted by obesity. For example, diet-induced obesity accelerates the age-associated decline of thymic function, resulting in reduced output of naïve T cells and TCR diversity (Yang et al., 2009b). Just like aging, diet-induced obesity induces the accumulation of adipocytes in the bone marrow (Ambrosi et al., 2017), which impairs hematopoiesis (Naveiras et al., 2009). Secondary lymphoid organ function is also affected by obesity: lymphatic transport, dendritic cell migration, and lymph node structure are reduced (Weitman et al., 2013), and murine dendritic cells stimulate T cell responses less effectively in obesity (James et al., 2012; Mauro et al., 2017). In addition, the prevalence of dysfunctional T cell populations is increased in obesity (Shirakawa et al., 2016; Spielmann et al., 2014; Wang et al., 2019) and higher frequencies of T cells expressing markers of antigen experience are present in the circulation of obese humans and animals (Mauro et al., 2017; Wang et al., 2019), similar to aged individuals. Despite these challenges, effective antigen-specific CD8^+^ T cell responses can be generated in obesity in certain contexts, such as infection of mice with lymphocytic choriomeningitis virus (LCMV) (Khan et al., 2014). Thus, disease context is a factor that affects CD8^+^ T cell-intrinsic function with obesity.

Recent studies have demonstrated that CD8^+^ T cell responses are impaired in the context of cancer with obesity. The growth of syngeneic subcutaneous B16 melanoma, 4T1 breast, and 3LL lung carcinoma tumors is accelerated in C57BL/6 mice fed a high-fat diet (HFD) for 4–5 months (Wang et al., 2019). This is associated with a dysfunctional CD8^+^ T cell phenotype, characterized by enhanced expression of co-inhibitory receptors and reduced expression of cytokines and the proliferation marker Ki-67. Although the interpretation of co-inhibitory receptor expression can be complicated, given that these receptors can be upregulated following T cell activation as well as during T cell exhaustion, the decreased expression of cytokines and Ki-67 points to a dysfunctional state. This defect is CD8^+^ T cell-intrinsic, as it is also present with ex vivo T cell activation. Another study also found a reduced anti-tumor CD8^+^ T cell response with diet-induced obesity in a murine breast cancer model (Zhang et al., 2020). There is some evidence, albeit limited, to suggest that the anti-tumor immune response is reduced in obese human patients with cancer. For example, reduced T cell infiltration was described in obese colorectal cancer patients, and increased expression of markers of T cell exhaustion, including co-inhibitory receptor PD-1, was seen in tumor-infiltrating T cells in obese melanoma patients (Wang et al., 2019).

### Anti-tumor T cell responses with dietary restriction

Dietary restriction has long been suggested to enhance anti-tumor immune function (Fernandes et al., 1976), and this may occur through both systemic and tumor-specific mechanisms. Dietary restriction encompasses various interventions, ranging from a reduction of caloric intake, for example by starvation or a fasting-mimicking diet (FMD), to a diet lacking in specific components, such as a low-protein diet (Katewa and Kapahi, 2010), or intermittent fasting (de Cabo and Mattson, 2019). Some of the metabolic consequences of dietary restriction can be induced pharmacologically by agents known as caloric restriction mimetics (CRMs). It is important to distinguish relatively mild dietary restriction regimens often used in studies of anti-tumor immunity from severely reduced dietary intake leading to malnutrition and lack of necessary nutrients. Malnutrition negatively impacts the immune system, including CD8^+^ T cells, in both animals and humans (Iyer et al., 2012; Rytter et al., 2014; Taylor et al., 2013).

Dietary restriction may improve T cell responses through several systemic mechanisms and can delay or prevent the decline in T cell immunity induced by aging (Figure 2). First, age-induced thymic involution can be decreased by long-term dietary restriction, thereby enhancing thymic output in aged mice (Yang et al., 2009a). Second, feeding of a calorically restricted diet for 13 to 18 years leads to preservation of T cell functions with age in non-human primates, in addition to increased naïve T cell numbers and TCR repertoire (Messaoudi et al., 2006). Finally, dietary restriction induces a reduction of memory T cells in secondary lymphoid organs and circulation but an accumulation in the bone marrow, resulting in increased protection from both infectious and tumor challenges (Collins et al., 2019).

In addition to systemic effects on T cell immunity in general, anti-tumor immunity is specifically improved with dietary restriction. For example, a short-term (48 hr) fast enhances the efficacy of chemotherapy in the murine fibrosarcoma model MCA205 in a T cell-dependent manner (Pietrocola et al., 2016). CRMs, including ATP citrate lyase inhibitor hydroxycitrate, reduce tumor growth in chemotherapy-treated mice similarly to short-term fasting, in a manner dependent on CD8^+^ T cells. These effects extend to murine breast, colorectal and lung cancer models (Pietrocola et al., 2016). Notably, there is little to no effect of fasting or CRM alone in this setting, implying that cell death induced by chemotherapy is needed to induce the anti-tumor immune response, which can then be boosted by dietary restriction. Another study demonstrated similar findings with the 4T1 breast cancer and B16 melanoma models, using a low calorie FMD in combination with chemotherapy (Di Biase et al., 2016). However, in contrast to the Pietrocola et al. study, they observed a significant benefit of FMD alone as well, albeit less pronounced than in combination with chemotherapy. Systemically, the numbers of common lymphoid progenitors (CLPs) in the bone marrow as well as circulating CD8^+^ T cells are increased by FMD (Di Biase et al., 2016). Another CRM, glycolysis inhibitor 2-deoxyglucose (2-DG), also promotes an anti-tumor T cell response in combination with chemotherapy, although this is likely primarily caused by an effect on the tumor cells themselves, since the combination of 2-DG and chemotherapy also enhances tumor cell death in vitro (Bénéteau et al., 2012). The fact that CRMs can replicate the benefits of dietary restriction suggests that the therapeutic targeting of these pathways to promote anti-tumor immunity may be feasible.

Finally, reducing dietary protein may also enhance anti-tumor immunity. Rubio-Patiño et al. found that a low-protein diet reduced tumor progression in murine lymphoma, colorectal adenocarcinoma and melanoma models in a CD8^+^ T cell-dependent manner (Rubio-Patiño et al., 2018). Restriction of specific amino acids may also impact anti-tumor immunity. For example, dietary methionine restriction, which promotes lifespan and metabolic health in mice (Ables et al., 2012; Miller et al., 2005), can have anti-cancer effects (Gao et al., 2019; Wanders et al., 2020) and impacts helper T cell responses (Wang et al., 2020). Accordingly, a shift in macrophage polarization accompanied by increased intratumoral CD8^+^ T cell numbers and GzmB expression occurs in mice fed a methionine-restricted diet (Orillion et al., 2018). The extent to which reduced intake of particular amino acids may affect the anti-tumor T cell response is an interesting topic for future investigations.

### Metabolism and signaling

Metabolism plays crucial roles in regulating T cell activation, differentiation, and function (Buck et al., 2017; Lim et al., 2020; O'Sullivan et al., 2019). When naïve T cells are activated by ligation of the TCR and costimulatory signals, they initiate a phase of rapid growth, differentiation, and proliferation (Smith-Garvin et al., 2009). This transition is accompanied by a switch from a quiescent metabolic state, characterized by mostly oxidative metabolism, to a highly metabolically active state, characterized by the use of anabolic pathways such as anaerobic glycolysis, glutaminolysis, and one-carbon metabolism (Chang et al., 2013; Ho et al., 2015; Ron-Harel et al., 2016; Wang et al., 2011). These pathways not only provide the molecules necessary for cell growth and proliferation, but also support activation and differentiation in different ways. For example, glycolysis directly impacts translation of IFN-γ mRNA (Chang et al., 2013), calcium signaling (Ho et al., 2015) and epigenetic state (Peng et al., 2016) in activated T cells.

In contrast to effector T cells, memory T cells and Tregs rely on oxidative metabolic pathways, including fatty acid oxidation (FAO) (Michalek et al., 2011; Pearce et al., 2009; van der Windt et al., 2012). In many cases, manipulating metabolism is sufficient to change the differentiation of T cells toward distinct phenotypes, indicating that certain metabolic pathways lie upstream of fate decisions in T cells (Buck et al., 2016; Michalek et al., 2011; Pearce et al., 2009; Pollizzi et al., 2016; Verbist et al., 2016).

**Figure 3.**
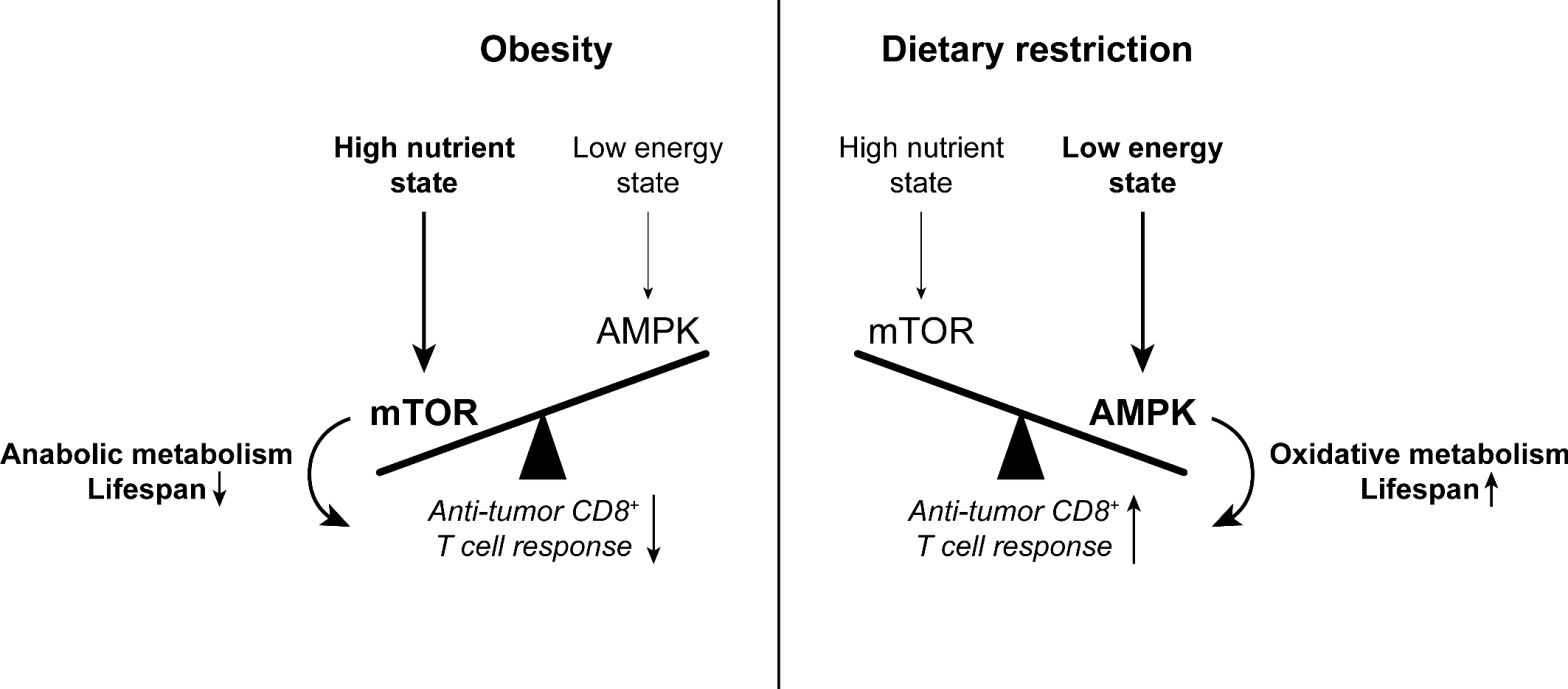
The balance between mTOR and AMPK signaling impacts anti-tumor immunity. High nutrient states induce mTOR signaling, which promotes anabolic metabolism and reduces lifespan. Conversely, low energy states induce AMPK signaling, which promotes oxidative metabolism and extends lifespan. While mTOR signaling is important for effector T cell responses, there is evidence to suggest that memory T cell-like oxidative metabolism may be more beneficial for the anti-tumor T cell response. Since mTOR signaling is enhanced with obesity while AMPK signaling is promoted by dietary restriction, altering the mTOR-AMPK balance may one way by which systemic metabolic state can affect CD8^+^ T cell function in cancer. AMPK, AMP-activated protein kinase. mTOR, mammalian target of rapamycin.

Several transcription factors and nutrient-sensitive signaling pathways coordinate the metabolic profiles of distinct T cell subsets. The anabolic metabolic program characteristic of effector T cells is orchestrated by mammalian target of rapamycin (mTOR), Myc, and hypoxia-inducible factor (HIF)−1α, which are engaged during T cell activation (Pollizzi and Powell, 2014). In contrast, memory T cell metabolism is promoted by AMP-activated protein kinase (AMPK) – a nutrient sensor that is activated during low energy conditions and opposes signaling through mTOR, which is activated by high nutrient levels (Pollizzi and Powell, 2014; Figure 3). Accordingly, memory T cell generation is promoted by metformin-induced AMPK activation as well as rapamycin-mediated mTOR inhibition (Araki et al., 2009; Pearce et al., 2009).

Although effector T cell responses have predominantly been associated with a shift toward anabolic metabolic pathways, a more oxidative metabolic profile may be beneficial for T cell survival and functionality in the context of a nutrient-depleted TME (see section on ‘Immune regulation in the tumor microenvironment’). Various interventions that promote oxidative, mitochondrial metabolic pathways in T cells enhance their anti-tumor function, for example promoting mitochondrial fusion, enhancing mitochondrial biogenesis, supplementing arginine, inducing fatty acid metabolism, and inhibiting glutamine metabolism (Buck et al., 2016; Chowdhury et al., 2018; Geiger et al., 2016; Leone et al., 2019; Scharping et al., 2016; Zhang et al., 2017).

Since the nutrient sensors mTOR and AMPK are affected by systemic metabolic state, an interesting question is how the balance between mTOR- and AMPK-mediated metabolism may impact anti-tumor T cell function downstream of systemic metabolism. While mTOR and AMPK are generally activated in different energy conditions, both signaling pathways are engaged during T cell activation (Pollizzi and Powell, 2014). Given the importance of mTOR signaling in T cell activation and acquisition of effector functions, the mTOR inhibitor rapamycin is generally viewed as immunosuppressive, and mTOR signaling has indeed been shown to support anti-tumor T cell responses (Chaoul et al., 2015; Pollizzi et al., 2015). However, rapamycin can also promote anti-tumor immunity in certain contexts, such as the MOC1 oral cancer model (Cash et al., 2015; Moore et al., 2016). On the other hand, T cell-intrinsic AMPK signaling is also required for T cell responses, including anti-tumor immunity, by promoting metabolic fitness and survival (Blagih et al., 2015; Rao et al., 2015), which may be of particular relevance in the context of a nutrient-depleted TME. Indeed, the AMPK activator metformin promotes anti-tumor T cell responses through several mechanisms, including AMPK activation in T cells (Eikawa et al., 2015), AMPK-dependent degradation of inhibitory ligand PD-L1 (Cha et al., 2018), and reduced oxygen consumption in tumor cells, likely resulting from AMPK-independent inhibition of the electron transport chain by metformin (Scharping et al., 2017). These findings show that the net result of the balance between effector function-promoting mTOR and metabolic fitness-promoting AMPK is context-dependent and can affect CD8^+^ T cell function both directly through T cell-intrinsic metabolic effects and indirectly through other cell types. Cellular nutrient sensors like mTOR and AMPK thus provide an example of how organismal metabolic state may interact with cellular metabolism and T cell function.

### Metabolism and signaling with aging

Aging is a process characterized by widespread metabolic and signaling changes impacting many of the pathways highlighted above (Barzilai et al., 2012), which may affect anti-tumor T cell responses. Specific metabolic changes considered hallmarks of aging are mitochondrial dysfunction and dysregulation of nutrient sensing (López-Otín et al., 2013). Underscoring the importance of metabolism in regulating lifespan and aging, inhibition of insulin, insulin-like growth factor (IGF)−1, or mTOR signaling increases lifespan, and so does enhancing AMPK signaling (López-Otín et al., 2016; López-Otín et al., 2013; Figure 3). mTOR signaling also impacts immunity in aging: inhibition of mTOR signaling improves hematopoiesis in aged mice as well as immune function in the contexts of vaccination and infection in elderly humans (Chen et al., 2009; Mannick et al., 2018; Mannick et al., 2014). Systemic inflammation is similarly associated with aging – often referred to as ‘inflammaging’ (López-Otín et al., 2013), and this contributes to aging-related decline, as evidenced for example by the observation that inhibition of pro-inflammatory NF-κB signaling can reduce aging phenotypes (Tilstra et al., 2012). Interestingly, recent research demonstrated that T cells may contribute to age-related inflammation and its metabolic complications, such as hyperglycemia (Bharath et al., 2020; Desdín-Micó et al., 2020; Lee et al., 2019), and engineered T cells can prevent pathology by clearing senescent cells (Amor et al., 2020). However, here we will focus on the inverse relationship: how do systemic changes associated with aging impact T cell responses, particularly in the context of cancer?

Cellular metabolic changes contribute to age-associated T cell dysfunction (Quinn et al., 2019; Ron-Harel et al., 2015). For example, aged T cells are characterized by mitochondrial dysfunction (Ron-Harel et al., 2018). This results in defective respiratory capacity and one-carbon metabolism, and supplementation with one-carbon metabolites, like formate and glycine, can improve aged T cell activation and survival (Ron-Harel et al., 2018). Both mitochondrial dysfunction and autophagy contribute to inflammatory phenotypes in aged T cells, and this can be improved by the AMPK activator metformin (Bharath et al., 2020). Mitogen-activated protein kinase (MAPK) signaling, through complex formation with a class of stress response proteins known as sestrins, directly contribute to aged T cell dysfunction (Lanna et al., 2017). It should be noted that the aforementioned studies were conducted in CD4^+^ T cells, and the effects in CD8^+^ T cells could thus be different, although signs of mitochondrial dysfunction have been seen in aged human CD8^+^ T cells as well (Moskowitz et al., 2017).

In some cases, different T cell subsets can display distinct metabolic phenotypes with age. For example, aged CD8^+^ Tvm cell were recently shown to have enhanced respiratory capacity despite being dysfunctional (Quinn et al., 2020). Furthermore, aged memory CD8^+^ T cells may have increased mTOR activity and glycolysis well as a reduced ability to engage in autophagy (Davenport et al., 2019; Henson et al., 2014), while newly activated aged CD4^+^ T cells fail to induce glycolysis as robustly as younger counterparts (Ron-Harel et al., 2018). Thus, while the exact metabolic properties of distinct T cell subsets in aging need to be further delineated, there is substantial evidence that metabolic dysregulation contributes to T cell dysfunction in aging.

### Metabolism and signaling with obesity

Like aging, obesity is characterized by profound systemic changes in both humans and animal models, including insulin resistance, dyslipidemia, and inflammation, collectively leading to the so-called metabolic syndrome (Barzilai et al., 2012; Huang, 2009). Adipose tissue inflammation plays a key role in the pathophysiology of metabolic syndrome, and adipose tissue secretion of adipokines, such as the satiety regulator leptin, and inflammatory cytokines into the circulation contributes to systemic inflammatory and metabolic dysregulation (Bremer and Jialal, 2013). While not the focus of this review, it is noteworthy that T cells also play an important role in the development of adipose tissue inflammation and its systemic sequelae. Specifically, infiltration of CD8^+^ and inflammatory CD4^+^ T cells into adipose tissue is increased with obesity, while CD4^+^ Tregs are reduced, contributing to adipose tissue inflammation and insulin resistance (Feuerer et al., 2009; Nishimura et al., 2009; Winer et al., 2009).

Obesity and metabolic syndrome are characterized by mTOR activation (Liu and Sabatini, 2020; Figure 3). mTOR hyperactivation contributes to development of the metabolic syndrome, including insulin resistance, as well as chronic inflammation, for example by promoting inflammatory cytokine secretion by cells of the innate immune system in the liver and adipose tissue (Jiang et al., 2014; Liu and Sabatini, 2020). In turn, chronic inflammation associated with obesity is thought to promote the dysfunctional T cell states described above (Shirakawa et al., 2016; Spielmann et al., 2014; Wang et al., 2019).

Given the importance of metabolism in regulating T cell fate and function, it is likely that the vast metabolic changes observed with obesity also affect T cell metabolism and function (Dyck and Lynch, 2018; Turbitt et al., 2020). Indeed, in the context of influenza infection with HFD feeding, C57BL/6 mouse CD8^+^ T cells display a shift toward oxidative metabolism accompanied by defective memory T cell responses (Rebeles et al., 2019). This finding highlights the complex context-dependent interactions between T cell metabolism, fate, and function, given that oxidative metabolism is generally thought to promote memory T cell development (Pearce et al., 2009; van der Windt et al., 2012). CD8^+^ T cells from obese BALB/c mice have increases in both spare respiratory capacity and glycolytic reserve, indicating a more general metabolic dysfunction (Turbitt et al., 2020). These findings contrast with the observed mitochondrial dysfunction in aged CD8^+^ T cells.

Increased circulating levels of the adipokine leptin with diet-induced obesity contribute to the observed dysfunction in CD8^+^ T cells in the context of cancer, as leptin receptor-deficient T cells mount a more effective anti-tumor response after adoptive transfer into HFD-fed recipients (Wang et al., 2019). This is an interesting finding, given that the roles of leptin, while complex and incompletely understood, are predominantly thought to promote T cell responses (Naylor and Petri, 2016). For example, leptin promotes proliferation, function, and glycolytic metabolism in CD4^+^ T cells (Gerriets et al., 2016; Lord et al., 1998; Saucillo et al., 2014). Leptin also enhances mitochondrial metabolism in CD8^+^ T cells, and oncolytic virus-mediated expression of leptin in the TME improved CD8^+ ^T cell function in a mouse model of melanoma (Rivadeneira et al., 2019). One potential explanation for this discrepancy is that leptin may have different effects in the context of lean compared to obese organisms due to chronic exposure to elevated leptin levels with obesity. This hypothesis is supported by the observations that intratumoral leptin delivery provides no benefit in obese mice, in contrast to controls (Rivadeneira et al., 2019), and that neutralization of leptin improves immunotherapy responsiveness in mice with diet-induced obesity (Murphy et al., 2018). Further supporting the notion that leptin may reduce CD8^+^ T cell anti-tumor function in the context of obesity, a recent study found that leptin contributes to CD8^+^ tumor-infiltrating lymphocyte (TIL) dysfunction in a spontaneous breast cancer model (Zhang et al., 2020). This occurs through a STAT3-mediated increase in FAO in CD8^+^ TILs, suggesting that FAO is harmful for the anti-tumor T cell response. Like for leptin, different studies have yielded opposing results regarding the impact of FAO on CD8^+^ TIL functionality. CD8^+^ T cells upregulate FAO in oxygen- and glucose-poor environments, like the TME, and promoting FAO through PPARα activation enhances CD8^+^ TIL function in melanoma models (Zhang et al., 2017) and murine colorectal adenocarcinoma models (Chowdhury et al., 2018). In all, the effects of FAO and leptin on anti-tumor CD8^+^ T cell function are complex and likely dependent on many environmental factors, and further studies are needed to delineate these.

In addition to leptin, circulating levels of free fatty acids and cholesterol are increased with obesity, and these too may impact CD8^+^ T cell function, although there is currently only circumstantial evidence to support this. Elevated fatty acid levels promote the differentiation of CD4^+^ T cells toward an effector-memory-like phenotype in obesity (Mauro et al., 2017) and suppress the NK cell-mediated anti-tumor response through the accumulation of intracellular lipids (Michelet et al., 2018). Lipid accumulation in the TME of a pancreatic cancer model led to intracellular lipid accumulation in CD8^+^ T cells, reducing their functionality (Manzo et al., 2020). Enabling the T cells to use these lipids for oxidation restored T cell function. Therefore, how CD8^+^ T cells handle the lipids in a particular context, i.e. intracellular accumulation versus use for FAO, may be a factor that determines how environmental lipids affect T cell function. Moreover, the cholesterol content of the cell membrane modulates CD8^+^ T cell function in tumors (Yang et al., 2016), but it is unclear whether this is significantly impacted by increased cholesterol levels in the circulation with obesity.

Another metabolic feature often associated with obesity is insulin resistance, which leads to increased plasma levels of both glucose and insulin. Hyperglycemia does not appear to affect CD8^+^ T cell function (Recino et al., 2017), but the effects of hyperinsulinemia on CD8^+^ T cells are not well-understood. T cells do express the insulin receptor and insulin signaling in T cells is required for their metabolic reprogramming upon activation and optimal anti-viral responsiveness (Fischer et al., 2017; Tsai et al., 2018), suggesting that T cells could be sensitive to systemic alterations in insulin signaling. Moreover, obesity-associated hyperinsulinemia inhibits IL-10-mediated immunosuppression by Tregs (Han et al., 2014). Activated T cells from obese human subjects bind less insulin, suggesting reduced expression of the insulin receptor in this condition (Helderman and Raskin, 1980). However, the functional consequences of obesity-associated alterations in insulin signaling on the anti-tumor CD8^+^ T cell response are currently unknown.

### Metabolism and signaling with dietary restriction

The biological effects of dietary restriction are in many ways, perhaps expectedly, opposite to those seen in obesity. Although human data are scarcer for dietary restriction than obesity, animal studies have suggested that dietary restriction may provide widespread benefits, including protection from various pathologies and increased lifespan (Fontana and Partridge, 2015; López-Otín et al., 2016; Mattison et al., 2017; McCay et al., 1935). Dietary restriction also reduces cancer incidence in animal studies (Colman et al., 2009; Michels and Ekbom, 2004; Ross and Bras, 1965; Rous, 1914; Weindruch and Walford, 1982) and may improve the efficacy of cancer therapies, including chemotherapy, radiotherapy and targeted therapies (Kopeina et al., 2017; O'Flanagan et al., 2017).

Prominent systemic metabolic consequences of dietary restriction include activation of AMPK and PPARα signaling as well as a reduction of mTOR signaling, due to low insulin, IGF-1 and glucose levels, together resulting in an increase in catabolic and mitochondrial metabolism (Kopeina et al., 2017; Martin-Montalvo and de Cabo, 2013; Figure 3). These metabolic changes are important, as reduced IGF-1 levels contribute to the anti-cancer effects of caloric restriction in mice (Nogueira et al., 2012). AMPK-mediated metabolism also counteracts inflammatory pathways, thus dampening age-related systemic inflammation and its consequences (O'Neill and Hardie, 2013).

As described above, effector T cell responses are associated with mTOR-mediated anabolic metabolism, whereas memory T cell development is supported by AMPK-promoted oxidative pathways. However, in many instances, interventions that induce oxidative, mitochondrial metabolism result in enhanced anti-tumor T cell function (Buck et al., 2016; Geiger et al., 2016; Leone et al., 2019; Scharping et al., 2016), and AMPK signaling can promote anti-tumor immunity (Cha et al., 2018; Eikawa et al., 2015; Rao et al., 2015). Dietary restriction also induces sirtuin 1 activity (Cohen et al., 2004), which can support anti-tumor function in CD4^+^ T cells (Chatterjee et al., 2018). Effector and memory T cell subsets may respond differently to such systemic metabolic alterations given their distinct metabolic dependencies. Indeed, memory T cells assume a metabolically quiescent state with reduced mTOR signaling during dietary restriction, resulting in increased protection in infection and tumors (Collins et al., 2019). Further work is needed to understand how systemic metabolic alterations with dietary restriction, shifting toward catabolic, AMPK-induced metabolism, may contribute to enhanced CD8^+^ T cell responses against tumors.

### Immune modulation in the tumor microenvironment

**Figure 4.**
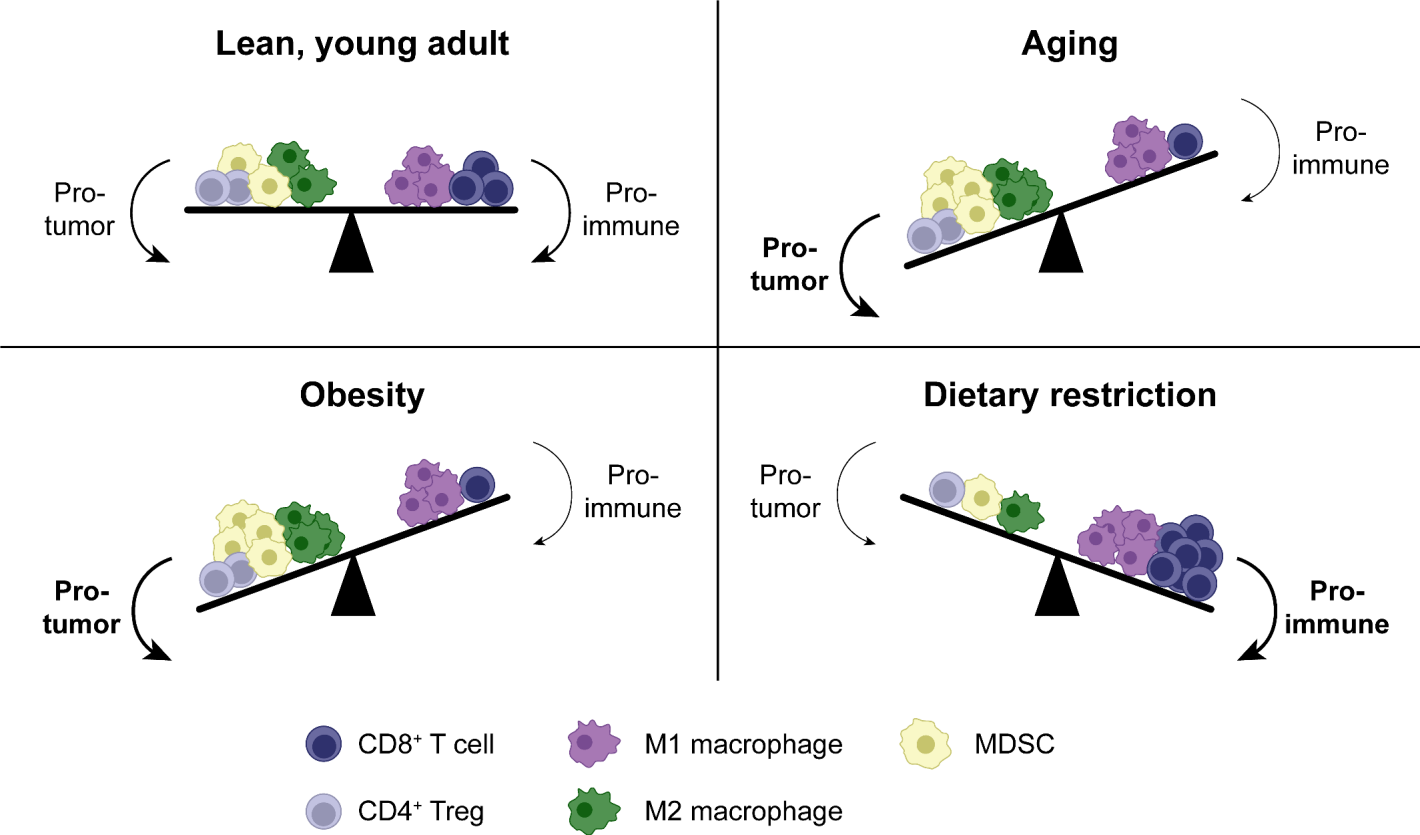
Shifts in the tumor immune infiltrate affect anti-tumor CD8^+^ T cell function in distinct systemic conditions. In addition to CD8^+^ T cells, the TME contains other immune populations, some of which are immunostimulatory (e.g. M1-polarized macrophages), while others are suppressive (e.g. M2-polarized macrophages, MDSCs and Tregs). In aging, enhanced MDSC numbers and potentially M2 macrophages contribute to immunosuppression, resulting in a reduced anti-tumor CD8^+^ T cell response. Tregs may be either increased or decreased with aging. Similar shifts in cellular populations in the TME exist with obesity. In dietary restriction, Treg and MDSC numbers are reduced while macrophage polarization is shifted toward the M1 phenotype, resulting in an increased anti-tumor CD8^+^ T cell response. MDSC, myeloid-derived suppressor cell. TME, tumor microenvironment. Treg, regulatory T cell.

Both cellular and metabolic factors contribute to the immunosuppressive nature of the TME. Cancer cells, Tregs, macrophages, MDSCs, and fibroblasts cultivate a highly immunosuppressive microenvironment, where CD8^+^ T cell responses are impaired by signaling through inhibitory receptors, suppressive cytokines, and reduced antigen presentation (Anderson et al., 2017; Figure 4). Metabolic challenges in the TME constitute another layer of immunosuppression (Lim et al., 2020). For example, T cells compete with tumor cells and other intratumoral cell populations for nutrients, which are limited by the tumor vasculature and high density of metabolically active tumor cells. This has been shown most specifically for glucose (Chang et al., 2015; Ho et al., 2015), but may apply to other nutrients as well. Accordingly, immunosuppressive cells in the TME express the enzymes arginase and indoleamine 2,3-dioxygenase, which degrade the amino acids arginine and tryptophan respectively, resulting in suppression of T cell responses (Rodriguez et al., 2004; Uyttenhove et al., 2003). Moreover, metabolic waste products, such as lactate, accumulate in the TME and suppress T cells (Brand et al., 2016). Finally, tumors often induce cell-intrinsic metabolic changes in T cells, like reduced mitochondrial mass and function, which impair their anti-tumor functionality (Kumar et al., 2020; Scharping et al., 2016).

### Immune modulation in the tumor microenvironment with aging

Aging alters the TME in various ways (Fane and Weeraratna, 2020). For example, the aged TME is characterized by increased immunosuppressive immune populations. Both Tregs and MDSCs have been implicated in enhanced immunosuppression in aged tumors (Dominguez and Lustgarten, 2008; Grizzle et al., 2007; Hurez et al., 2012; Sekido et al., 2019; Sharma et al., 2006), although the literature on Tregs is inconsistent, as some studies have described reduced Tregs numbers in tumors in aged animals (Kugel et al., 2018). As for the mechanisms underlying increased MDSC infiltration in aged tumors, local senescent stromal cells, most notably fibroblasts, promote MDSC accumulation in the TME through the secretion of inflammatory cytokines like IL-6 (Ruhland et al., 2016). M2 macrophages, another immunosuppressive myeloid population, have been less studied in this context, but they are also enriched in lymphoid tissues of aged mice (Jackaman et al., 2013) and might be enhanced in tumors as well (Figure 4).

Aged fibroblasts can also perform additional immunosuppressive roles. For example, mitochondrial dysfunction in cancer-associated fibroblasts may lead to enhanced secretion of reactive oxygen species and lactate (Balliet et al., 2011), thereby making the TME less permissive to T cell function. Moreover, fibroblast dysfunction contributes to a decrease in extracellular matrix (ECM) integrity with age, which, in addition to promoting cancer metastasis, likely impairs CD8^+^ T cell infiltration (Kaur et al., 2019). The aged microenvironment can also impact cancer cell-intrinsic signaling pathways, such as Wnt signaling (Kaur et al., 2016), which in turn might affect immune infiltration (Spranger et al., 2015). However, the tumor cell-intrinsic signaling and metabolic changes with aging are generally not well-understood.

### Immune modulation in the tumor microenvironment with obesity

Little is currently known about how the systemic metabolic alterations associated with obesity impact the metabolic conditions in the TME locally. Changes to the intratumoral cellular populations are somewhat better understood. Most notably and similar to aging, the TME contains higher numbers of immunosuppressive myeloid cells with obesity, including MDSCs (Clements et al., 2018; Hale et al., 2015; Turbitt et al., 2019), neutrophils (Incio et al., 2016), and dysfunctional dendritic cells (James et al., 2012) in multiple cancer models. These populations contribute to cancer progression, metastasis, and suppression of CD8^+^ T cell function (Clements et al., 2018; Incio et al., 2016; James et al., 2012; Norian et al., 2009; Quail et al., 2017). M2-polarized macrophage numbers are increased in human breast adipose tissue with obesity, suggesting that these may also play a role in the context of cancer (Springer et al., 2019). In contrast, Treg infiltration in tumors is similar in animals with diet-induced obesity compared to controls (Wang et al., 2019), suggesting that enhanced immunosuppression in the obese TME is largely mediated by myeloid populations (Figure 4).

### Immune modulation in the tumor microenvironment with dietary restriction

Some of the beneficial effect of dietary restriction on anti-tumor immunity is likely caused by tumor cell-intrinsic metabolic changes. For example, a low-protein diet induces tumoral endoplasmic reticulum (ER) stress pathways, mediated by inositol-requiring enzyme (IRE)1α, and retinoic acid-inducible gene (RIG)-I activation, which may induce an immunostimulatory cytokine response (Rubio-Patiño et al., 2018). Tumor cell-intrinsic changes also stimulate the anti-tumor immune response by reducing immunosuppressive populations in the TME, such as Tregs. For example, dietary restriction or a CRM enhances the efficacy of chemotherapy and anti-tumor CD8^+^ T cell response in a manner dependent on cancer cell autophagy, which may deplete Tregs from the tumor bed by releasing ATP (Pietrocola et al., 2016). FMD also prevents Treg accumulation in the TME, which is partially dependent on downregulation of heme oxygenase (HO)−1 in tumors (Di Biase et al., 2016).

In addition to Tregs, dietary restriction also reduces myeloid cell-mediated immunosuppression in the TME. MDSC numbers are reduced in tumors in mice with dietary restriction, correlating with enhanced CD8^+^ T cell infiltration (Turbitt et al., 2019), and there is evidence to suggest that shifting away from mTOR-mediated and toward AMPK-mediated metabolism impairs the suppressive capacity of MDSCs (Deng et al., 2018; Salminen et al., 2019). Low-protein diet shifts the balance of tumor-associated macrophages away from the immunosuppressive M2 phenotype and toward the inflammatory M1 phenotype (Orillion et al., 2018). Thus, tumor cell-intrinsic metabolic changes and a reduction in suppressive immune populations appear to be the main drivers for the enhanced anti-tumor CD8^+^ T cell response seen with dietary restriction (Figure 4).

### Cancer immunotherapies

Cancer immunotherapeutic strategies include immune checkpoint blockade (ICB), adoptive cell therapy, cancer vaccines, and engagement of innate (e.g., Toll-like receptors) or adaptive (e.g. OX40) immunostimulatory receptors. Immune checkpoint blockade (ICB) and adoptive cell therapy are now FDA-approved cancer immunotherapies. With ICB, antibodies are used to prevent the binding of inhibitory receptors on T cells, such as CTLA-4 and PD-1, to their ligands. CTLA-4-targeting antibodies were approved for melanoma treatment in 2011, making them the first approved form of ICB for cancer therapy (Baumeister et al., 2016). Anti-PD-1 therapies were approved for melanoma in 2014 and PD-1 pathway blockade is now approved for many other cancer types, including solid tumors with a high degree of microsatellite instability or DNA mismatch repair deficiency regardless of anatomical location or tissue of origin (Boyiadzis et al., 2018; Waldman et al., 2020). An exciting feature of ICB therapies is that they induce very long-lasting responses in some patients (Ledford, 2016). However, many patients currently do not respond to ICB, and innovative strategies to provide the therapeutic benefits of ICB to more patients are therefore highly sought after (Park et al., 2018).

Another therapeutic strategy to induce an anti-tumor T cell response is the administration of T cells that can recognize cancer antigens. There are two main adoptive T cell therapy approaches. First, T cells can be isolated from a tumor and expanded ex vivo; this approach has had some success in melanoma (Rosenberg and Restifo, 2015). Second, T cells can be transduced with a TCR or a chimeric antigen receptor (CAR) specific for a cancer antigen. CAR T cell therapies have been most efficacious in hematological cancers by targeting the B cell antigen CD19, while success in solid tumors has been limited so far (Jacoby et al., 2019; June et al., 2018).

### Cancer immunotherapies with aging

Several immunotherapeutic modalities, such as engagement of immunostimulatory receptors (e.g. TLRs or OX40) and cancer vaccination strategies (using DNA, tumor cells or dendritic cells), are less effective in aged animals (Castro et al., 2009; Dominguez and Lustgarten, 2008; Grolleau-Julius et al., 2008; Provinciali et al., 2003; Provinciali et al., 2000; Ruby and Weinberg, 2009). Fewer T cells are recovered following adoptive transfer of young T cells into aged compared to young tumor-bearing mice (Farazi et al., 2014), suggesting that adoptive cell therapies may also be less effective with aging, in a manner dependent on the aged host environment. These results have contributed to the general notion of decreased anti-tumor immune function with age.

**Figure 5.**
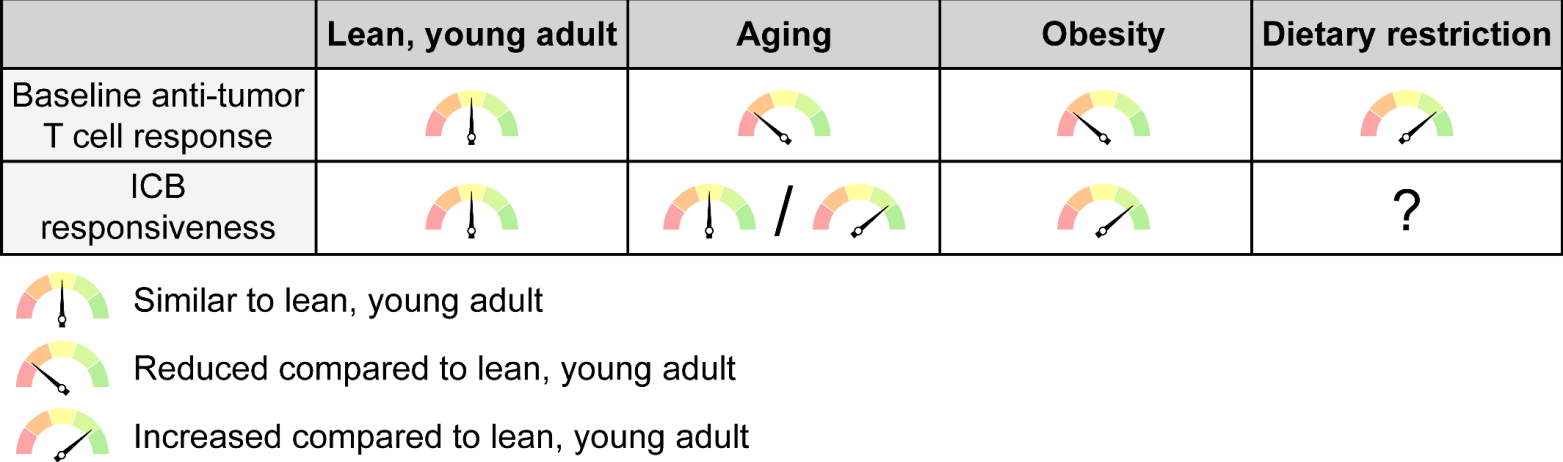
The net effects of systemic conditions on anti-tumor immunity and ICB responsiveness. Aging and obesity lead to a reduced anti-tumor T cell response compared to a young, lean adult at baseline, i.e. without immunotherapy, while dietary restriction enhances the baseline anti-tumor immune response. However, the efficacy of ICB therapy is intact or even enhanced with aging and mostly increased with obesity, although this may not apply to all cancer types and patient subsets. ICB responsiveness with dietary restriction has not been sufficiently studied to know whether this is altered. ICB, immune checkpoint blockade.

In contrast, most available data suggest that the efficacy of ICB is intact in aged human cancer patients and in animal tumor models (Figure 5). Kugel et al. found that responsiveness to anti-PD-1 was even enhanced in older human melanoma patients and aged mice, and this was associated with increased CD8^+^ T cell infiltration (Kugel et al., 2018). Others have also found good ICB responsiveness in elderly patients with melanoma (Ben-Betzalel et al., 2019; Betof et al., 2017) or non-small cell lung cancer (Lichtenstein et al., 2019), and across multiple cancer types (Corbaux et al., 2019). Overall, most human studies show at least similar efficacy of anti-CTLA-4 and anti-PD-(L)1 therapies in older compared to younger patients (Huang et al., 2020; Pawelec, 2019; Poropatich et al., 2017).

In murine studies, the responsiveness to ICB varies with cancer type and the immunotherapy used. Anti-CTLA-4, anti-PD-1 and anti-PD-L1 monotherapies are all highly effective in aged animals with an oral cancer model (Sekido et al., 2019). Aged and young mice respond similarly to anti-PD-L1 immunotherapy in a hematological malignancy model (Mirza et al., 2010). However, in the B16 melanoma model, anti-PD-1 is equally effective between aged and young mice, whereas anti-CTLA-4 works less well and anti-PD-L1 does not work at all (Padrón et al., 2018). Responsiveness to anti-CTLA-4 and anti-PD-L1 is also reduced in aged breast cancer models, but efficacy can be improved by enhancing interferon signaling in the TME (Sceneay et al., 2019). Thus, while ICB appears to remain efficacious with aging in mice and humans, further studies are needed to delineate the (determinants of) efficacy of different immunotherapeutic modalities in aged populations.

### Cancer immunotherapies with obesity

Surprisingly, although obesity reduces the anti-tumor immune response, the efficacy of ICB cancer immunotherapy is enhanced in both animal models and human obesity in multiple cancer types (McQuade et al., 2018; Wang et al., 2019; Xu et al., 2019; Figure 5). However, not all studies have found enhanced efficacy of ICB immunotherapy in mice with diet-induced obesity (Murphy et al., 2018). This may be explained by several differences, including the mouse strain (BALB/c versus C57/BL6), cancer model (renal cell carcinoma versus melanoma and lung carcinoma models) and modalities of immunotherapy (anti-CTLA-4 combined with other immunostimulants versus anti-PD-1) (Murphy et al., 2018; Wang et al., 2019). The discrepancy in results obtained with these different animal models correlates with human data, where obesity improves the efficacy of ICB in patients with melanoma and non-small cell lung cancer, but not renal cell carcinoma (Xu et al., 2019). Sex may also be a relevant factor, since some studies have found that better outcomes after ICB with obesity are mostly driven by male patients (McQuade et al., 2018; Naik et al., 2019), although a meta-analysis found that they are independent of sex (Xu et al., 2019). Further studies are needed to determine which patient and cancer subsets display improved outcomes upon ICB therapy with obesity.

The increase in responsiveness to cancer immunotherapy with obesity in many cancer types suggests that obesity-associated immunosuppressive factors can be overcome with the removal of inhibitory signals like PD-(L)1 signaling. It is currently unknown whether the enhanced efficacy of ICB in obese compared to non-obese subjects can be explained by obesity-related systemic alterations that directly promote T cell function, or by indirect factors, such as tumor cells being more prone to immune attack. Finally, the findings of ICB responsiveness with obesity likely do not translate to all types of cancer immunotherapy, as other modalities have shown decreased functionality (James et al., 2012). This suggests that, like with aging, ICB responsiveness may be better than other immunotherapies in obesity.

### Cancer immunotherapies with dietary restriction

Very little information is available on responsiveness to cancer immunotherapy with dietary restriction. In one study, low-protein diet did not change anti-tumor immunity alone, but it did enhance responsiveness to anti-PD-1 in a mouse model of renal cell cancer (Orillion et al., 2018). This contrasts with a study discussed above, in which low-protein diet alone reduced tumor progression in murine lymphoma, colorectal adenocarcinoma and melanoma models (Rubio-Patiño et al., 2018). Dietary restriction improves the efficacy of OX40 agonist immunotherapy in murine tumors as well as activation of CD4^+^, but not CD8^+^, T cells (Farazi et al., 2014). The limited data available from animal studies suggest that there is no paradoxical reduction of ICB efficacy with dietary restriction, as might have been feared based on the data described above for aging and diet-induced obesity, but more studies are needed.

## Concluding remarks

While many outstanding questions remain, age and systemic metabolic state impact anti-tumor CD8^+^ T cell responses. Systemic and TME-specific factors in the host environment as well as CD8^+^ T cell-intrinsic factors play a role. There are striking similarities in the changes observed with aging and obesity: both induce a chronic inflammatory state, reduce naïve CD8^+^ T cell generation, promote dysfunctional T cell states, and increase immunosuppressive myeloid cell populations in tumors. Together, these factors significantly reduce the anti-tumor CD8^+^ T cell response. In contrast, dietary restriction promotes anti-tumor immunity by opposing most of these age- and obesity-related mechanisms. Dietary restriction also causes cancer cell-intrinsic metabolic changes, such as induction of autophagy and ER stress, which benefit the anti-tumor T cell response. A dietary restriction-induced systemic shift toward AMPK-mediated oxidative metabolism likely contributes to the development of an environment that is more conducive to anti-tumor CD8^+^ T cell function by reducing systemic inflammation and improving the TME cellular landscape. Additionally, enhanced AMPK signaling in CD8^+^ T cells themselves may promote their survival and function in the TME.

The parallels between aging and obesity, and how they contrast with dietary restriction, are further emphasized by research looking at a combination of these conditions. For example, the observed increase in expression of PD-1 and other exhaustion markers on T cells in melanoma tumors with high body weight was more pronounced in patients over 60 years of age (Wang et al., 2019). Moreover, dietary restriction can restore some of the age-related decline in anti-tumor function of CD4^+^ T cells and responsiveness to OX40 immunotherapy (Farazi et al., 2014). Aged and obese mice display a similar immune dysregulation, leading to a harmful cytokine storm upon administration of immunotherapy consisting of a costimulatory receptor CD40 agonist combined with IL-2, which caloric restriction protects against (Mirsoian et al., 2014). These findings suggest that aging and obesity activate common pathways to impair the anti-tumor immune response, and these can be counteracted by dietary restriction.

The observation that responsiveness to ICB immunotherapies, like anti-PD-1, is intact or even enhanced in aged or obese individuals is surprising. Typically, having a better pre-existing anti-tumor CD8^+^ T cell response, as evidenced by intratumoral CD8^+^ T cell infiltration, increases the chances of responding to ICB (Ji et al., 2012). The mechanisms underlying this paradox in the context of age and obesity are unclear. Perhaps some of the dysfunctional T cell populations in aging and obesity are more prone to (re)activation by blockade of coinhibitory receptor signaling. If so, that would also explain why, in contrast to ICB, other immunotherapy modalities yield reduced responses with age and obesity in animal models. Intratumoral exhausted CD8^+^ T cells can be divided into several subpopulations including ‘terminally exhausted’ and ‘progenitor exhausted’ populations, the former having higher cytolytic function and the latter being more important for responsiveness to anti-PD-1 therapy (Beltra et al., 2020; Hudson et al., 2019; Im et al., 2016; Miller et al., 2019). It would be interesting to investigate whether an altered distribution of intratumoral CD8^+^ T cells between these populations with aging or obesity could contribute to the observed paradox of reduced baseline anti-tumor immunity with intact ICB responses.

Despite much progress over recent years, many important questions remain unanswered. First, how conditions of obesity or dietary restriction impact nutrient availability in the TME is largely a black box. Dietary changes can alter the metabolic composition of the TME (Maddocks et al., 2017; Sullivan et al., 2019), but this has not been analyzed for HFD or dietary restriction. Given the limited blood supply and high local metabolic rates in a tumor, alterations in metabolite concentrations in the circulation may not be proportionally reflected in the TME (Sullivan et al., 2019). Moreover, diet can change cancer cell metabolism (Bose et al., 2020; Lien and Vander Heiden, 2019), further shaping the metabolic conditions in the TME and in some cases benefitting specific intratumoral cell populations (Kumagai et al., 2020). Second, it is largely unknown what the intracellular fates of nutrients in the TME are and how these may differ between cell types. Third, there are various systemic conditions in addition to the ones discussed here for which the effects on anti-tumor immunity are unknown, and it would be interesting to explore those. Fourth, since mouse research studying obesity often involves the feeding of a HFD, the question arises whether the observed effects are due to the diet itself or the obese state. Delineating the contributions of diet and obese state to both systemic and TME-specific metabolic alterations will be an exciting avenue for future studies with potential therapeutic implications. And finally, preclinical findings with dietary restriction, suggesting enhanced anti-tumor T cell responses, look promising. It needs to be determined whether combination of dietary restriction and CRMs with immunotherapies can boost anti-tumor immunity, and some such trials are already underway (Lévesque et al., 2019). To further facilitate this, it would be important to assess which exact dietary interventions are most beneficial in which context and what metabolic changes underly any therapeutic benefit.

Research investigating the impact of age and metabolic state on both cancer biology and anti-tumor immunity also has methodological implications and highlights shortcomings of the currently available technologies. For one, it may be important to use aged and/or metabolically unhealthy animals for investigations into the anti-tumor immune response, in addition to the lean, young animals that are most commonly used, to better model cancer patients and improve the translatability of any findings. In that regard, it is noteworthy that there is a high degree of congruency between existing human and animal data concerning anti-tumor immunity and ICB responsiveness with age, obesity, and dietary restriction. Moreover, despite the value and practicality of using implantable tumor models for animal studies, they have some limitations, including (1) they do not assess effects of the ‘agedness’ of cancer cells themselves, and (2) they study cancer progression, whereas most human studies quantify cancer incidence. There may be value in using animal models of spontaneous tumor growth to assess how age and diet alter immune surveillance, although there are challenges to this approach, as genetic cancer models are typically not very immunogenic. Finally, it remains very challenging to determine the activity intracellular metabolic pathways in distinct cell populations in a tissue, for example a tumor, in vivo. Mass spectrometry-based approaches are complicated, given that only limited CD8^+^ T cell numbers can be obtained from a tumor and extensive processing and sorting procedures are required for their isolation, which are bound to impact metabolite pool sizes. Recently, several groups have made progress in this area, for example using in vivo infusion of isotopically labeled glucose to assess T cell metabolism (Ma et al., 2019) or tracing isotopically labeled nutrients into macromolecules with a slow turnover rate in distinct intratumoral cell populations, specifically cancer cells and fibroblasts (Lau et al., 2020). Others have developed advanced flow cytometric strategies for reading out metabolic characteristics at a single-cell level (Ahl et al., 2020).

Cancer represents a huge public health burden, and the cancer risk factors of old age and obesity are increasing in prevalence. It is therefore increasingly critical to consider these factors in the design of cancer immunotherapies. Expanding our knowledge of the macroenvironmental determinants of anti-tumor immunity should reveal new ways to extend the benefit of cancer immunotherapies to more patients.

## References

[bib1] Ables GP, Perrone CE, Orentreich D, Orentreich N (2012). Methionine-restricted C57BL/6J mice are resistant to diet-induced obesity and insulin resistance but have low bone density. PLOS ONE.

[bib2] Ahl PJ, Hopkins RA, Xiang WW, Au B, Kaliaperumal N, Fairhurst AM, Connolly JE (2020). Met-Flow, a strategy for single-cell metabolic analysis highlights dynamic changes in immune subpopulations. Communications Biology.

[bib3] Ambrosi TH, Scialdone A, Graja A, Gohlke S, Jank AM, Bocian C, Woelk L, Fan H, Logan DW, Schürmann A, Saraiva LR, Schulz TJ (2017). Adipocyte accumulation in the bone marrow during obesity and aging impairs stem Cell-Based hematopoietic and bone regeneration. Cell Stem Cell.

[bib4] Amor C, Feucht J, Leibold J, Ho YJ, Zhu C, Alonso-Curbelo D, Mansilla-Soto J, Boyer JA, Li X, Giavridis T, Kulick A, Houlihan S, Peerschke E, Friedman SL, Ponomarev V, Piersigilli A, Sadelain M, Lowe SW (2020). Senolytic CAR T cells reverse senescence-associated pathologies. Nature.

[bib5] Anderson KG, Stromnes IM, Greenberg PD (2017). Obstacles posed by the tumor microenvironment to T cell Activity: A Case for Synergistic Therapies. Cancer Cell.

[bib6] Araki K, Turner AP, Shaffer VO, Gangappa S, Keller SA, Bachmann MF, Larsen CP, Ahmed R (2009). mTOR regulates memory CD8 Tcell differentiation. Nature.

[bib7] Arias E, Xu J (2019). United States Life Tables, 2017.

[bib8] Bains I, Thiébaut R, Yates AJ, Callard R (2009). Quantifying thymic export: combining models of naive T cell proliferation and TCR excision circle dynamics gives an explicit measure of thymic output. The Journal of Immunology.

[bib9] Balliet RM, Capparelli C, Guido C, Pestell TG, Martinez-Outschoorn UE, Lin Z, Whitaker-Menezes D, Chiavarina B, Pestell RG, Howell A, Sotgia F, Lisanti MP (2011). Mitochondrial oxidative stress in cancer-associated fibroblasts drives lactate production, promoting breast Cancer tumor growth. Cell Cycle.

[bib10] Barber DL, Wherry EJ, Masopust D, Zhu B, Allison JP, Sharpe AH, Freeman GJ, Ahmed R (2006). Restoring function in exhausted CD8 T cells during chronic viral infection. Nature.

[bib11] Barzilai N, Huffman DM, Muzumdar RH, Bartke A (2012). The critical role of metabolic pathways in aging. Diabetes.

[bib12] Baumeister SH, Freeman GJ, Dranoff G, Sharpe AH (2016). Coinhibitory pathways in immunotherapy for Cancer. Annual Review of Immunology.

[bib13] Beerman I, Bhattacharya D, Zandi S, Sigvardsson M, Weissman IL, Bryder D, Rossi DJ (2010). Functionally distinct hematopoietic stem cells modulate hematopoietic lineage potential during aging by a mechanism of clonal expansion. PNAS.

[bib14] Beltra JC, Manne S, Abdel-Hakeem MS, Kurachi M, Giles JR, Chen Z, Casella V, Ngiow SF, Khan O, Huang YJ, Yan P, Nzingha K, Xu W, Amaravadi RK, Xu X, Karakousis GC, Mitchell TC, Schuchter LM, Huang AC, Wherry EJ (2020). Developmental relationships of four exhausted CD8^+ ^T Cell Subsets Reveals Underlying Transcriptional and Epigenetic Landscape Control Mechanisms. Immunity.

[bib15] Ben-Betzalel G, Steinberg-Silman Y, Stoff R, Asher N, Shapira-Frommer R, Schachter J, Markel G (2019). Immunotherapy comes of age in octagenarian and nonagenarian metastatic melanoma patients. European Journal of Cancer.

[bib16] Bénéteau M, Zunino B, Jacquin MA, Meynet O, Chiche J, Pradelli LA, Marchetti S, Cornille A, Carles M, Ricci JE (2012). Combination of glycolysis inhibition with chemotherapy results in an antitumor immune response. PNAS.

[bib17] Betof AS, Nipp RD, Giobbie-Hurder A, Johnpulle RAN, Rubin K, Rubinstein SM, Flaherty KT, Lawrence DP, Johnson DB, Sullivan RJ (2017). Impact of age on outcomes with immunotherapy for patients with melanoma. The Oncologist.

[bib18] Bharath LP, Agrawal M, McCambridge G, Nicholas DA, Hasturk H, Liu J, Jiang K, Liu R, Guo Z, Deeney J, Apovian CM, Snyder-Cappione J, Hawk GS, Fleeman RM, Pihl RMF, Thompson K, Belkina AC, Cui L, Proctor EA, Kern PA, Nikolajczyk BS (2020). Metformin enhances autophagy and normalizes mitochondrial function to alleviate Aging-Associated inflammation. Cell Metabolism.

[bib19] Blackburn SD, Shin H, Haining WN, Zou T, Workman CJ, Polley A, Betts MR, Freeman GJ, Vignali DA, Wherry EJ (2009). Coregulation of CD8+ T cell exhaustion by multiple inhibitory receptors during chronic viral infection. Nature Immunology.

[bib20] Blagih J, Coulombe F, Vincent EE, Dupuy F, Galicia-Vázquez G, Yurchenko E, Raissi TC, van der Windt GJ, Viollet B, Pearce EL, Pelletier J, Piccirillo CA, Krawczyk CM, Divangahi M, Jones RG (2015). The energy sensor AMPK regulates T cell metabolic adaptation and effector responses in vivo. Immunity.

[bib21] Bose S, Allen AE, Locasale JW (2020). The molecular link from diet to Cancer cell metabolism. Molecular Cell.

[bib22] Boyiadzis MM, Kirkwood JM, Marshall JL, Pritchard CC, Azad NS, Gulley JL (2018). Significance and implications of FDA approval of pembrolizumab for biomarker-defined disease. Journal for ImmunoTherapy of Cancer.

[bib23] Brand A, Singer K, Koehl GE, Kolitzus M, Schoenhammer G, Thiel A, Matos C, Bruss C, Klobuch S, Peter K, Kastenberger M, Bogdan C, Schleicher U, Mackensen A, Ullrich E, Fichtner-Feigl S, Kesselring R, Mack M, Ritter U, Schmid M, Blank C, Dettmer K, Oefner PJ, Hoffmann P, Walenta S, Geissler EK, Pouyssegur J, Villunger A, Steven A, Seliger B, Schreml S, Haferkamp S, Kohl E, Karrer S, Berneburg M, Herr W, Mueller-Klieser W, Renner K, Kreutz M (2016). LDHA-Associated Lactic Acid Production Blunts Tumor Immunosurveillance by T and NK Cells. Cell Metabolism.

[bib24] Bremer AA, Jialal I (2013). Adipose tissue dysfunction in nascent metabolic syndrome. Journal of Obesity.

[bib25] Buck MD, O'Sullivan D, Klein Geltink RI, Curtis JD, Chang CH, Sanin DE, Qiu J, Kretz O, Braas D, van der Windt GJ, Chen Q, Huang SC, O'Neill CM, Edelson BT, Pearce EJ, Sesaki H, Huber TB, Rambold AS, Pearce EL (2016). Mitochondrial dynamics controls T cell fate through metabolic programming. Cell.

[bib26] Buck MD, Sowell RT, Kaech SM, Pearce EL (2017). Metabolic instruction of immunity. Cell.

[bib27] Burnet M (1957). Cancer: a biological approach. British Medical Journal.

[bib28] Cash H, Shah S, Moore E, Caruso A, Uppaluri R, Van Waes C, Allen C (2015). mTOR and MEK1/2 inhibition differentially modulate tumor growth and the immune microenvironment in syngeneic models of oral cavity Cancer. Oncotarget.

[bib29] Castro F, Leal B, Denny A, Bahar R, Lampkin S, Reddick R, Lu S, Gravekamp C (2009). Vaccination with Mage-b DNA induces CD8 T-cell responses at young but not old age in mice with metastatic breast Cancer. British Journal of Cancer.

[bib30] Cha JH, Yang WH, Xia W, Wei Y, Chan LC, Lim SO, Li CW, Kim T, Chang SS, Lee HH, Hsu JL, Wang HL, Kuo CW, Chang WC, Hadad S, Purdie CA, McCoy AM, Cai S, Tu Y, Litton JK, Mittendorf EA, Moulder SL, Symmans WF, Thompson AM, Piwnica-Worms H, Chen CH, Khoo KH, Hung MC (2018). Metformin promotes antitumor immunity via Endoplasmic-Reticulum-Associated degradation of PD-L1. Molecular Cell.

[bib31] Chang CH, Curtis JD, Maggi LB, Faubert B, Villarino AV, O'Sullivan D, Huang SC, van der Windt GJ, Blagih J, Qiu J, Weber JD, Pearce EJ, Jones RG, Pearce EL (2013). Posttranscriptional control of T cell effector function by aerobic glycolysis. Cell.

[bib32] Chang CH, Qiu J, O'Sullivan D, Buck MD, Noguchi T, Curtis JD, Chen Q, Gindin M, Gubin MM, van der Windt GJ, Tonc E, Schreiber RD, Pearce EJ, Pearce EL (2015). Metabolic competition in the tumor microenvironment is a driver of Cancer progression. Cell.

[bib33] Chaoul N, Fayolle C, Desrues B, Oberkampf M, Tang A, Ladant D, Leclerc C (2015). Rapamycin impairs antitumor CD8+ T-cell responses and Vaccine-Induced tumor eradication. Cancer Research.

[bib34] Chaplin DD (2010). Overview of the immune response. Journal of Allergy and Clinical Immunology.

[bib35] Chatterjee S, Daenthanasanmak A, Chakraborty P, Wyatt MW, Dhar P, Selvam SP, Fu J, Zhang J, Nguyen H, Kang I, Toth K, Al-Homrani M, Husain M, Beeson G, Ball L, Helke K, Husain S, Garrett-Mayer E, Hardiman G, Mehrotra M, Nishimura MI, Beeson CC, Bupp MG, Wu J, Ogretmen B, Paulos CM, Rathmell J, Yu XZ, Mehrotra S (2018). CD38-NAD^+^Axis regulates immunotherapeutic Anti-Tumor T cell response. Cell Metabolism.

[bib36] Chen C, Liu Y, Liu Y, Zheng P (2009). mTOR regulation and therapeutic rejuvenation of aging hematopoietic stem cells. Science Signaling.

[bib37] Chiu BC, Martin BE, Stolberg VR, Chensue SW (2013). Cutting edge: central memory CD8 T cells in aged mice are virtual memory cells. The Journal of Immunology.

[bib38] Chowdhury PS, Chamoto K, Kumar A, Honjo T (2018). PPAR-Induced fatty acid oxidation in T cells increases the number of Tumor-Reactive CD8^+^ T Cells and Facilitates Anti-PD-1 Therapy. Cancer Immunology Research.

[bib39] Clements VK, Long T, Long R, Figley C, Smith DMC, Ostrand-Rosenberg S (2018). Frontline science: high fat diet and leptin promote tumor progression by inducing myeloid-derived suppressor cells. Journal of Leukocyte Biology.

[bib40] Cohen HY, Miller C, Bitterman KJ, Wall NR, Hekking B, Kessler B, Howitz KT, Gorospe M, de Cabo R, Sinclair DA (2004). Calorie restriction promotes mammalian cell survival by inducing the SIRT1 deacetylase. Science.

[bib41] Collins N, Han SJ, Enamorado M, Link VM, Huang B, Moseman EA, Kishton RJ, Shannon JP, Dixit D, Schwab SR, Hickman HD, Restifo NP, McGavern DB, Schwartzberg PL, Belkaid Y (2019). The bone marrow protects and optimizes immunological memory during dietary restriction. Cell.

[bib42] Colman RJ, Anderson RM, Johnson SC, Kastman EK, Kosmatka KJ, Beasley TM, Allison DB, Cruzen C, Simmons HA, Kemnitz JW, Weindruch R (2009). Caloric restriction delays disease onset and mortality in rhesus monkeys. Science.

[bib43] Cooper MD, Peterson RD, Good RA (1965). Delineation of the thymic and bursal lymphoid systems in the chicken. Nature.

[bib44] Corbaux P, Maillet D, Boespflug A, Locatelli-Sanchez M, Perier-Muzet M, Duruisseaux M, Kiakouama-Maleka L, Dalle S, Falandry C, Péron J (2019). Older and younger patients treated with immune checkpoint inhibitors have similar outcomes in real-life setting. European Journal of Cancer.

[bib45] Davenport B, Eberlein J, van der Heide V, Jhun K, Nguyen TT, Victorino F, Trotta A, Chipuk J, Yi Z, Zhang W, Clambey ET, Scott DK, Homann D (2019). Aging of antiviral CD8^+^memory T cells fosters increased survival, metabolic adaptations, and lymphoid tissue homing. The Journal of Immunology.

[bib46] de Cabo R, Mattson MP (2019). Effects of intermittent fasting on health, aging, and disease. New England Journal of Medicine.

[bib47] Decker WK, da Silva RF, Sanabria MH, Angelo LS, Guimarães F, Burt BM, Kheradmand F, Paust S (2017). Cancer immunotherapy: historical perspective of a clinical revolution and emerging preclinical animal models. Frontiers in Immunology.

[bib48] Decman V, Laidlaw BJ, Dimenna LJ, Abdulla S, Mozdzanowska K, Erikson J, Ertl HC, Wherry EJ (2010). Cell-intrinsic defects in the proliferative response of antiviral memory CD8 T cells in aged mice upon secondary infection. The Journal of Immunology.

[bib49] Decman V, Laidlaw BJ, Doering TA, Leng J, Ertl HC, Goldstein DR, Wherry EJ (2012). Defective CD8 T cell responses in aged mice are due to quantitative and qualitative changes in virus-specific precursors. The Journal of Immunology.

[bib50] Deng Y, Yang J, Luo F, Qian J, Liu R, Zhang D, Yu H, Chu Y (2018). mTOR-mediated glycolysis contributes to the enhanced suppressive function of murine tumor-infiltrating monocytic myeloid-derived suppressor cells. Cancer Immunology, Immunotherapy.

[bib51] Desdín-Micó G, Soto-Heredero G, Aranda JF, Oller J, Carrasco E, Gabandé-Rodríguez E, Blanco EM, Alfranca A, Cussó L, Desco M, Ibañez B, Gortazar AR, Fernández-Marcos P, Navarro MN, Hernaez B, Alcamí A, Baixauli F, Mittelbrunn M (2020). T cells with dysfunctional mitochondria induce multimorbidity and premature senescence. Science.

[bib52] Di Biase S, Lee C, Brandhorst S, Manes B, Buono R, Cheng CW, Cacciottolo M, Martin-Montalvo A, de Cabo R, Wei M, Morgan TE, Longo VD (2016). Fasting-Mimicking diet reduces HO-1 to promote T Cell-Mediated Tumor Cytotoxicity. Cancer Cell.

[bib53] Dominguez AL, Lustgarten J (2008). Implications of aging and self-tolerance on the generation of immune and antitumor immune responses. Cancer Research.

[bib54] Dong H, Strome SE, Salomao DR, Tamura H, Hirano F, Flies DB, Roche PC, Lu J, Zhu G, Tamada K, Lennon VA, Celis E, Chen L (2002). Tumor-associated B7-H1 promotes T-cell apoptosis: a potential mechanism of immune evasion. Nature Medicine.

[bib55] Dunn GP, Bruce AT, Ikeda H, Old LJ, Schreiber RD (2002). Cancer immunoediting: from immunosurveillance to tumor escape. Nature Immunology.

[bib56] Dyck L, Lynch L (2018). Cancer, obesity and immunometabolism – Connecting the dots. Cancer Letters.

[bib57] Ehrlich P (1909). Über den jetzigen stand der karzinomforschung. Nederlands Tijdschrift Voor Geneeskunde.

[bib58] Eikawa S, Nishida M, Mizukami S, Yamazaki C, Nakayama E, Udono H (2015). Immune-mediated antitumor effect by type 2 diabetes drug, metformin. PNAS.

[bib59] Esensten JH, Helou YA, Chopra G, Weiss A, Bluestone JA (2016). CD28 costimulation: from mechanism to therapy. Immunity.

[bib60] Fane M, Weeraratna AT (2020). How the ageing microenvironment influences tumour progression. Nature Reviews Cancer.

[bib61] Farazi M, Nguyen J, Goldufsky J, Linnane S, Lukaesko L, Weinberg AD, Ruby CE (2014). Caloric restriction maintains OX40 agonist-mediated tumor immunity and CD4 T cell priming during aging. Cancer Immunology, Immunotherapy.

[bib62] Farhood B, Najafi M, Mortezaee K (2019). CD8^+^ cytotoxic T lymphocytes in Cancer immunotherapy: a review. Journal of Cellular Physiology.

[bib63] Fernandes G, Yunis EJ, Good RA (1976). Suppression of adenocarcinoma by the immunological consequences of calorie restriction. Nature.

[bib64] Feuerer M, Herrero L, Cipolletta D, Naaz A, Wong J, Nayer A, Lee J, Goldfine AB, Benoist C, Shoelson S, Mathis D (2009). Lean, but not obese, fat is enriched for a unique population of regulatory T cells that affect metabolic parameters. Nature Medicine.

[bib65] Filaci G, Fenoglio D, Fravega M, Ansaldo G, Borgonovo G, Traverso P, Villaggio B, Ferrera A, Kunkl A, Rizzi M, Ferrera F, Balestra P, Ghio M, Contini P, Setti M, Olive D, Azzarone B, Carmignani G, Ravetti JL, Torre G, Indiveri F (2007). CD8^+^ CD28- T regulatory lymphocytes inhibiting T cell proliferative and cytotoxic functions infiltrate human cancers. Journal of Immunology.

[bib66] Fischer HJ, Sie C, Schumann E, Witte AK, Dressel R, van den Brandt J, Reichardt HM (2017). The insulin receptor plays a critical role in T cell function and adaptive immunity. The Journal of Immunology.

[bib67] Fontana L, Partridge L (2015). Promoting health and longevity through diet: from model organisms to humans. Cell.

[bib68] Gao X, Sanderson SM, Dai Z, Reid MA, Cooper DE, Lu M, Richie JP, Ciccarella A, Calcagnotto A, Mikhael PG, Mentch SJ, Liu J, Ables G, Kirsch DG, Hsu DS, Nichenametla SN, Locasale JW (2019). Dietary methionine influences therapy in mouse Cancer models and alters human metabolism. Nature.

[bib69] Geiger R, Rieckmann JC, Wolf T, Basso C, Feng Y, Fuhrer T, Kogadeeva M, Picotti P, Meissner F, Mann M, Zamboni N, Sallusto F, Lanzavecchia A (2016). L-Arginine modulates T cell metabolism and enhances survival and Anti-tumor activity. Cell.

[bib70] Gerriets VA, Danzaki K, Kishton RJ, Eisner W, Nichols AG, Saucillo DC, Shinohara ML, MacIver NJ (2016). Leptin directly promotes T-cell glycolytic metabolism to drive effector T-cell differentiation in a mouse model of autoimmunity. European Journal of Immunology.

[bib71] Goronzy JJ, Weyand CM (2019). Mechanisms underlying T cell ageing. Nature Reviews Immunology.

[bib72] Graham JB, Graham RM (1959). The effect of vaccine on Cancer patients. Plastic and Reconstructive Surgery.

[bib73] Grizzle WE, Xu X, Zhang S, Stockard CR, Liu C, Yu S, Wang J, Mountz JD, Zhang HG (2007). Age-related increase of tumor susceptibility is associated with myeloid-derived suppressor cell mediated suppression of T cell cytotoxicity in recombinant inbred BXD12 mice. Mechanisms of Ageing and Development.

[bib74] Grolleau-Julius A, Garg MR, Mo R, Stoolman LL, Yung RL (2006). Effect of aging on bone Marrow-Derived murine CD11c+CD4-CD8 - Dendritic Cell Function. The Journals of Gerontology Series A: Biological Sciences and Medical Sciences.

[bib75] Grolleau-Julius A, Harning EK, Abernathy LM, Yung RL (2008). Impaired dendritic cell function in aging leads to defective antitumor immunity. Cancer Research.

[bib76] Hale M, Itani F, Buchta CM, Wald G, Bing M, Norian LA (2015). Obesity triggers enhanced MDSC accumulation in murine renal tumors via elevated local production of CCL2. PLOS ONE.

[bib77] Han JM, Patterson SJ, Speck M, Ehses JA, Levings MK (2014). Insulin Inhibits IL-10–Mediated Regulatory T Cell Function: Implications for Obesity. The Journal of Immunology.

[bib78] Helderman JH, Raskin P (1980). The T Lymphocyte insulin Receptor in Diabetes and Obesity: An Intrinsic Binding Defect. Diabetes.

[bib79] Henson SM, Lanna A, Riddell NE, Franzese O, Macaulay R, Griffiths SJ, Puleston DJ, Watson AS, Simon AK, Tooze SA, Akbar AN (2014). p38 signaling inhibits mTORC1-independent autophagy in senescent human CD8⁺ T cells. Journal of Clinical Investigation.

[bib80] Ho PC, Bihuniak JD, Macintyre AN, Staron M, Liu X, Amezquita R, Tsui YC, Cui G, Micevic G, Perales JC, Kleinstein SH, Abel ED, Insogna KL, Feske S, Locasale JW, Bosenberg MW, Rathmell JC, Kaech SM (2015). Phosphoenolpyruvate is a metabolic checkpoint of Anti-tumor T cell responses. Cell.

[bib81] Hotamisligil GS (2017). Foundations of immunometabolism and implications for metabolic health and disease. Immunity.

[bib82] Huang PL (2009). A comprehensive definition for metabolic syndrome. Disease Models & Mechanisms.

[bib83] Huang XZ, Gao P, Song YX, Sun JX, Chen XW, Zhao JH, Wang ZN (2020). Efficacy of immune checkpoint inhibitors and age in Cancer patients. Immunotherapy.

[bib84] Hudson WH, Gensheimer J, Hashimoto M, Wieland A, Valanparambil RM, Li P, Lin JX, Konieczny BT, Im SJ, Freeman GJ, Leonard WJ, Kissick HT, Ahmed R (2019). Proliferating transitory T cells with an Effector-like transcriptional signature emerge from PD-1^+^ Stem-like CD8^+^ T Cells during Chronic Infection. Immunity.

[bib85] Hurez V, Daniel BJ, Sun L, Liu AJ, Ludwig SM, Kious MJ, Thibodeaux SR, Pandeswara S, Murthy K, Livi CB, Wall S, Brumlik MJ, Shin T, Zhang B, Curiel TJ (2012). Mitigating age-related immune dysfunction heightens the efficacy of tumor immunotherapy in aged mice. Cancer Research.

[bib86] Im SJ, Hashimoto M, Gerner MY, Lee J, Kissick HT, Burger MC, Shan Q, Hale JS, Lee J, Nasti TH, Sharpe AH, Freeman GJ, Germain RN, Nakaya HI, Xue HH, Ahmed R (2016). Defining CD8+ T cells that provide the proliferative burst after PD-1 therapy. Nature.

[bib87] Incio J, Liu H, Suboj P, Chin SM, Chen IX, Pinter M, Ng MR, Nia HT, Grahovac J, Kao S, Babykutty S, Huang Y, Jung K, Rahbari NN, Han X, Chauhan VP, Martin JD, Kahn J, Huang P, Desphande V, Michaelson J, Michelakos TP, Ferrone CR, Soares R, Boucher Y, Fukumura D, Jain RK (2016). Obesity-Induced inflammation and desmoplasia promote pancreatic Cancer progression and resistance to chemotherapy. Cancer Discovery.

[bib88] Islami F, Goding Sauer A, Gapstur SM, Jemal A (2018). Proportion of Cancer cases attributable to excess body weight by US state, 2011-2015. JAMA Oncology.

[bib89] Iyer SS, Chatraw JH, Tan WG, Wherry EJ, Becker TC, Ahmed R, Kapasi ZF (2012). Protein energy malnutrition impairs homeostatic proliferation of memory CD8 T cells. The Journal of Immunology.

[bib90] Jackaman C, Radley-Crabb HG, Soffe Z, Shavlakadze T, Grounds MD, Nelson DJ (2013). Targeting macrophages rescues age-related immune deficiencies in C57BL/6J geriatric mice. Aging Cell.

[bib91] Jackaman C, Gardner JK, Tomay F, Spowart J, Crabb H, Dye DE, Fox S, Proksch S, Metharom P, Dhaliwal SS, Nelson DJ (2019). CD8^+^ cytotoxic T cell responses to dominant tumor-associated antigens are profoundly weakened by aging yet subdominant responses retain functionality and expand in response to chemotherapy. OncoImmunology.

[bib92] Jacoby E, Shahani SA, Shah NN (2019). Updates on CAR T-cell therapy in B-cell malignancies. Immunological Reviews.

[bib93] James BR, Tomanek-Chalkley A, Askeland EJ, Kucaba T, Griffith TS, Norian LA (2012). Diet-induced obesity alters dendritic cell function in the presence and absence of tumor growth. The Journal of Immunology.

[bib94] Ji RR, Chasalow SD, Wang L, Hamid O, Schmidt H, Cogswell J, Alaparthy S, Berman D, Jure-Kunkel M, Siemers NO, Jackson JR, Shahabi V (2012). An immune-active tumor microenvironment favors clinical response to ipilimumab. Cancer Immunology, Immunotherapy : CII.

[bib95] Jiang H, Westerterp M, Wang C, Zhu Y, Ai D (2014). Macrophage mTORC1 disruption reduces inflammation and insulin resistance in obese mice. Diabetologia.

[bib96] June CH, O’Connor RS, Kawalekar OU, Ghassemi S, Milone MC (2018). CAR T cell immunotherapy for human cancer. Science.

[bib97] Katewa SD, Kapahi P (2010). Dietary restriction and aging, 2009. Aging Cell.

[bib98] Kaur A, Webster MR, Marchbank K, Behera R, Ndoye A, Kugel CH, Dang VM, Appleton J, O'Connell MP, Cheng P, Valiga AA, Morissette R, McDonnell NB, Ferrucci L, Kossenkov AV, Meeth K, Tang HY, Yin X, Wood WH, Lehrmann E, Becker KG, Flaherty KT, Frederick DT, Wargo JA, Cooper ZA, Tetzlaff MT, Hudgens C, Aird KM, Zhang R, Xu X, Liu Q, Bartlett E, Karakousis G, Eroglu Z, Lo RS, Chan M, Menzies AM, Long GV, Johnson DB, Sosman J, Schilling B, Schadendorf D, Speicher DW, Bosenberg M, Ribas A, Weeraratna AT (2016). sFRP2 in the aged microenvironment drives melanoma metastasis and therapy resistance. Nature.

[bib99] Kaur A, Ecker BL, Douglass SM, Kugel CH, Webster MR, Almeida FV, Somasundaram R, Hayden J, Ban E, Ahmadzadeh H, Franco-Barraza J, Shah N, Mellis IA, Keeney F, Kossenkov A, Tang HY, Yin X, Liu Q, Xu X, Fane M, Brafford P, Herlyn M, Speicher DW, Wargo JA, Tetzlaff MT, Haydu LE, Raj A, Shenoy V, Cukierman E, Weeraratna AT (2019). Remodeling of the collagen matrix in aging skin promotes melanoma metastasis and affects immune cell motility. Cancer Discovery.

[bib100] Khan SH, Hemann EA, Legge KL, Norian LA, Badovinac VP (2014). Diet-induced obesity does not impact the generation and maintenance of primary memory CD8 T cells. The Journal of Immunology.

[bib101] Khandekar MJ, Cohen P, Spiegelman BM (2011). Molecular mechanisms of Cancer development in obesity. Nature Reviews Cancer.

[bib102] Kochanek KD, Murphy SL, Xu J, Arias E (2019). Deaths: Final Data for 2017.

[bib103] Kopeina GS, Senichkin VV, Zhivotovsky B (2017). Caloric restriction - A promising anti-cancer approach: from molecular mechanisms to clinical trials. Biochimica Et Biophysica Acta (BBA) - Reviews on Cancer.

[bib104] Kugel CH, Douglass SM, Webster MR, Kaur A, Liu Q, Yin X, Weiss SA, Darvishian F, Al-Rohil RN, Ndoye A, Behera R, Alicea GM, Ecker BL, Fane M, Allegrezza MJ, Svoronos N, Kumar V, Wang DY, Somasundaram R, Hu-Lieskovan S, Ozgun A, Herlyn M, Conejo-Garcia JR, Gabrilovich D, Stone EL, Nowicki TS, Sosman J, Rai R, Carlino MS, Long GV, Marais R, Ribas A, Eroglu Z, Davies MA, Schilling B, Schadendorf D, Xu W, Amaravadi RK, Menzies AM, McQuade JL, Johnson DB, Osman I, Weeraratna AT (2018). Age correlates with response to Anti-PD1, reflecting Age-Related differences in intratumoral effector and regulatory T-Cell populations. Clinical Cancer Research.

[bib105] Kumagai S, Togashi Y, Sakai C, Kawazoe A, Kawazu M, Ueno T, Sato E, Kuwata T, Kinoshita T, Yamamoto M, Nomura S, Tsukamoto T, Mano H, Shitara K, Nishikawa H (2020). An oncogenic alteration creates a microenvironment that promotes tumor progression by conferring a metabolic advantage to regulatory T cells. Immunity.

[bib106] Kumar V, Patel S, Tcyganov E, Gabrilovich DI (2016). The nature of Myeloid-Derived suppressor cells in the tumor microenvironment. Trends in Immunology.

[bib107] Kumar A, Chamoto K, Chowdhury PS, Honjo T (2020). Tumors attenuating the mitochondrial activity in T cells escape from PD-1 blockade therapy. eLife.

[bib108] Lages CS, Lewkowich I, Sproles A, Wills-Karp M, Chougnet C (2010). Partial restoration of T-cell function in aged mice by in vitro blockade of the PD-1/ PD-L1 pathway. Aging Cell.

[bib109] Lanna A, Gomes DC, Muller-Durovic B, McDonnell T, Escors D, Gilroy DW, Lee JH, Karin M, Akbar AN (2017). A sestrin-dependent Erk-Jnk-p38 MAPK activation complex inhibits immunity during aging. Nature Immunology.

[bib110] Lau AN, Li Z, Danai LV, Westermark AM, Darnell AM, Ferreira R, Gocheva V, Sivanand S, Lien EC, Sapp KM, Mayers JR, Biffi G, Chin CR, Davidson SM, Tuveson DA, Jacks T, Matheson NJ, Yilmaz O, Vander Heiden MG (2020). Dissecting cell-type-specific metabolism in pancreatic ductal adenocarcinoma. eLife.

[bib111] Lauby-Secretan B, Scoccianti C, Loomis D, Grosse Y, Bianchini F, Straif K, International Agency for Research on Cancer Handbook Working Group (2016). Body fatness and Cancer--viewpoint of the IARC working group. New England Journal of Medicine.

[bib112] Ledford H (2016). Cocktails for Cancer with a measure of immunotherapy. Nature.

[bib113] Lee YH, Kim SR, Han DH, Yu HT, Han YD, Kim JH, Kim SH, Lee CJ, Min BH, Kim DH, Kim KH, Cho JW, Lee WW, Shin EC, Park S (2019). Senescent T cells predict the development of hyperglycemia in humans. Diabetes.

[bib114] Leone RD, Zhao L, Englert JM, Sun IM, Oh MH, Sun IH, Arwood ML, Bettencourt IA, Patel CH, Wen J, Tam A, Blosser RL, Prchalova E, Alt J, Rais R, Slusher BS, Powell JD (2019). Glutamine blockade induces divergent metabolic programs to overcome tumor immune evasion. Science.

[bib115] Lévesque S, Pol JG, Ferrere G, Galluzzi L, Zitvogel L, Kroemer G (2019). Trial watch: dietary interventions for Cancer therapy. OncoImmunology.

[bib116] Li M, Yao D, Zeng X, Kasakovski D, Zhang Y, Chen S, Zha X, Li Y, Xu L (2019). Age related human T cell subset evolution and senescence. Immunity & Ageing.

[bib117] Lichtenstein MRL, Nipp RD, Muzikansky A, Goodwin K, Anderson D, Newcomb RA, Gainor JF (2019). Impact of age on outcomes with immunotherapy in patients with Non-Small cell lung Cancer. Journal of Thoracic Oncology.

[bib118] Lien EC, Vander Heiden MG (2019). A framework for examining how diet impacts tumour metabolism. Nature Reviews Cancer.

[bib119] Lighter J, Phillips M, Hochman S, Sterling S, Johnson D, Francois F, Stachel A (2020). Obesity in patients younger than 60 years is a risk factor for COVID-19 hospital admission. Clinical Infectious Diseases.

[bib120] Lim AR, Rathmell WK, Rathmell JC (2020). The tumor microenvironment as a metabolic barrier to effector T cells and immunotherapy. eLife.

[bib121] Liu GY, Sabatini DM (2020). mTOR at the nexus of nutrition, growth, ageing and disease. Nature Reviews Molecular Cell Biology.

[bib122] López-Otín C, Blasco MA, Partridge L, Serrano M, Kroemer G (2013). The hallmarks of aging. Cell.

[bib123] López-Otín C, Galluzzi L, Freije JMP, Madeo F, Kroemer G (2016). Metabolic control of longevity. Cell.

[bib124] Lord GM, Matarese G, Howard JK, Baker RJ, Bloom SR, Lechler RI (1998). Leptin modulates the T-cell immune response and reverses starvation-induced immunosuppression. Nature.

[bib125] Lustgarten J, Dominguez AL, Thoman M (2004). Aged mice develop protective antitumor immune responses with appropriate costimulation. The Journal of Immunology.

[bib126] Ma EH, Verway MJ, Johnson RM, Roy DG, Steadman M, Hayes S, Williams KS, Sheldon RD, Samborska B, Kosinski PA, Kim H, Griss T, Faubert B, Condotta SA, Krawczyk CM, DeBerardinis RJ, Stewart KM, Richer MJ, Chubukov V, Roddy TP, Jones RG (2019). Metabolic profiling using stable isotope tracing reveals distinct patterns of glucose utilization by physiologically activated CD8+ T cells. Immunity.

[bib127] Maddocks ODK, Athineos D, Cheung EC, Lee P, Zhang T, van den Broek NJF, Mackay GM, Labuschagne CF, Gay D, Kruiswijk F, Blagih J, Vincent DF, Campbell KJ, Ceteci F, Sansom OJ, Blyth K, Vousden KH (2017). Modulating the therapeutic response of tumours to dietary serine and glycine starvation. Nature.

[bib128] Mannick JB, Del Giudice G, Lattanzi M, Valiante NM, Praestgaard J, Huang B, Lonetto MA, Maecker HT, Kovarik J, Carson S, Glass DJ, Klickstein LB (2014). mTOR inhibition improves immune function in the elderly. Science Translational Medicine.

[bib129] Mannick JB, Morris M, Hockey HP, Roma G, Beibel M, Kulmatycki K, Watkins M, Shavlakadze T, Zhou W, Quinn D, Glass DJ, Klickstein LB (2018). TORC1 inhibition enhances immune function and reduces infections in the elderly. Science Translational Medicine.

[bib130] Manzo T, Prentice BM, Anderson KG, Raman A, Schalck A, Codreanu GS, Nava Lauson CB, Tiberti S, Raimondi A, Jones MA, Reyzer M, Bates BM, Spraggins JM, Patterson NH, McLean JA, Rai K, Tacchetti C, Tucci S, Wargo JA, Rodighiero S, Clise-Dwyer K, Sherrod SD, Kim M, Navin NE, Caprioli RM, Greenberg PD, Draetta G, Nezi L (2020). Accumulation of long-chain fatty acids in the tumor microenvironment drives dysfunction in intrapancreatic CD8+ T cells. Journal of Experimental Medicine.

[bib131] Martin-Montalvo A, de Cabo R (2013). Mitochondrial metabolic reprogramming induced by calorie restriction. Antioxidants & Redox Signaling.

[bib132] Mattison JA, Colman RJ, Beasley TM, Allison DB, Kemnitz JW, Roth GS, Ingram DK, Weindruch R, de Cabo R, Anderson RM (2017). Caloric restriction improves health and survival of rhesus monkeys. Nature Communications.

[bib133] Mauro C, Smith J, Cucchi D, Coe D, Fu H, Bonacina F, Baragetti A, Cermenati G, Caruso D, Mitro N, Catapano AL, Ammirati E, Longhi MP, Okkenhaug K, Norata GD, Marelli-Berg FM (2017). Obesity-Induced metabolic stress leads to biased effector memory CD4^+^T Cell Differentiation via PI3K p110δ-Akt-Mediated Signals. Cell Metabolism.

[bib134] McCarthy EF (2006). The toxins of William B. Coley and the treatment of bone and soft-tissue sarcomas. The Iowa Orthopaedic Journal.

[bib135] McCay CM, Crowell MF, Maynard LA (1935). The effect of retarded growth upon the length of life span and upon the ultimate body size. The Journal of Nutrition.

[bib136] McQuade JL, Daniel CR, Hess KR, Mak C, Wang DY, Rai RR, Park JJ, Haydu LE, Spencer C, Wongchenko M, Lane S, Lee DY, Kaper M, McKean M, Beckermann KE, Rubinstein SM, Rooney I, Musib L, Budha N, Hsu J, Nowicki TS, Avila A, Haas T, Puligandla M, Lee S, Fang S, Wargo JA, Gershenwald JE, Lee JE, Hwu P, Chapman PB, Sosman JA, Schadendorf D, Grob JJ, Flaherty KT, Walker D, Yan Y, McKenna E, Legos JJ, Carlino MS, Ribas A, Kirkwood JM, Long GV, Johnson DB, Menzies AM, Davies MA (2018). Association of body-mass index and outcomes in patients with metastatic melanoma treated with targeted therapy, immunotherapy, or chemotherapy: a retrospective, multicohort analysis. The Lancet Oncology.

[bib137] Messaoudi I, Warner J, Fischer M, Park B, Hill B, Mattison J, Lane MA, Roth GS, Ingram DK, Picker LJ, Douek DC, Mori M, Nikolich-Zugich J (2006). Delay of T cell senescence by caloric restriction in aged long-lived nonhuman primates. PNAS.

[bib138] Michalek RD, Gerriets VA, Jacobs SR, Macintyre AN, MacIver NJ, Mason EF, Sullivan SA, Nichols AG, Rathmell JC (2011). Cutting edge: distinct glycolytic and lipid oxidative metabolic programs are essential for effector and regulatory CD4^+^T cell subsets. The Journal of Immunology.

[bib139] Michelet X, Dyck L, Hogan A, Loftus RM, Duquette D, Wei K, Beyaz S, Tavakkoli A, Foley C, Donnelly R, O'Farrelly C, Raverdeau M, Vernon A, Pettee W, O'Shea D, Nikolajczyk BS, Mills KHG, Brenner MB, Finlay D, Lynch L (2018). Metabolic reprogramming of natural killer cells in obesity limits antitumor responses. Nature Immunology.

[bib140] Michels KB, Ekbom A (2004). Caloric restriction and incidence of breast Cancer. Jama.

[bib141] Miller JF, Mitchell GF, Weiss NS (1967). Cellular basis of the immunological defects in thymectomized mice. Nature.

[bib142] Miller RA, Buehner G, Chang Y, Harper JM, Sigler R, Smith-Wheelock M (2005). Methionine-deficient diet extends mouse lifespan, slows immune and lens aging, alters glucose, T4, IGF-I and insulin levels, and increases hepatocyte MIF levels and stress resistance. Aging Cell.

[bib143] Miller BC, Sen DR, Al Abosy R, Bi K, Virkud YV, LaFleur MW, Yates KB, Lako A, Felt K, Naik GS, Manos M, Gjini E, Kuchroo JR, Ishizuka JJ, Collier JL, Griffin GK, Maleri S, Comstock DE, Weiss SA, Brown FD, Panda A, Zimmer MD, Manguso RT, Hodi FS, Rodig SJ, Sharpe AH, Haining WN (2019). Subsets of exhausted CD8^+^ T cells differentially mediate tumor control and respond to checkpoint blockade. Nature Immunology.

[bib144] Miller JS, Lanier LL (2019). Natural killer cells in Cancer immunotherapy. Annual Review of Cancer Biology.

[bib145] Mirsoian A, Bouchlaka MN, Sckisel GD, Chen M, Pai CC, Maverakis E, Spencer RG, Fishbein KW, Siddiqui S, Monjazeb AM, Martin B, Maudsley S, Hesdorffer C, Ferrucci L, Longo DL, Blazar BR, Wiltrout RH, Taub DD, Murphy WJ (2014). Adiposity induces lethal cytokine storm after systemic administration of stimulatory immunotherapy regimens in aged mice. Journal of Experimental Medicine.

[bib146] Mirza N, Duque MA, Dominguez AL, Schrum AG, Dong H, Lustgarten J (2010). B7-H1 expression on old CD8^+^ T cells negatively regulates the activation of immune responses in aged animals. The Journal of Immunology.

[bib147] Moore EC, Cash HA, Caruso AM, Uppaluri R, Hodge JW, Van Waes C, Allen CT (2016). Enhanced tumor control with combination mTOR and PD-L1 inhibition in syngeneic oral cavity cancers. Cancer Immunology Research.

[bib148] Moskowitz DM, Zhang DW, Hu B, Le Saux S, Yanes RE, Ye Z, Buenrostro JD, Weyand CM, Greenleaf WJ, Goronzy JJ (2017). Epigenomics of human CD8 T cell differentiation and aging. Science Immunology.

[bib149] Murphy KA, James BR, Sjaastad FV, Kucaba TA, Kim H, Brincks EL, Chua SC, Wilber A, Griffith TS (2018). Cutting edge: elevated leptin during Diet-Induced obesity reduces the efficacy of tumor immunotherapy. The Journal of Immunology.

[bib150] Murray PJ (2017). Macrophage polarization. Annual Review of Physiology.

[bib151] Naik GS, Waikar SS, Johnson AEW, Buchbinder EI, Haq R, Hodi FS, Schoenfeld JD, Ott PA (2019). Complex inter-relationship of body mass index, gender and serum creatinine on survival: exploring the obesity paradox in melanoma patients treated with checkpoint inhibition. Journal for ImmunoTherapy of Cancer.

[bib152] Nakamura K, Smyth MJ (2020). Myeloid immunosuppression and immune checkpoints in the tumor microenvironment. Cellular & Molecular Immunology.

[bib153] Naveiras O, Nardi V, Wenzel PL, Hauschka PV, Fahey F, Daley GQ (2009). Bone-marrow adipocytes as negative regulators of the haematopoietic microenvironment. Nature.

[bib154] Naylor C, Petri WA (2016). Leptin regulation of immune responses. Trends in Molecular Medicine.

[bib155] Nikolich-Žugich J, Li G, Uhrlaub JL, Renkema KR, Smithey MJ (2012). Age-related changes in CD8 T cell homeostasis and immunity to infection. Seminars in Immunology.

[bib156] Nishimura H, Nose M, Hiai H, Minato N, Honjo T (1999). Development of Lupus-like Autoimmune Diseases by Disruption of the PD-1 Gene Encoding an ITIM Motif-Carrying Immunoreceptor. Immunity.

[bib157] Nishimura S, Manabe I, Nagasaki M, Eto K, Yamashita H, Ohsugi M, Otsu M, Hara K, Ueki K, Sugiura S, Yoshimura K, Kadowaki T, Nagai R (2009). CD8+ effector T cells contribute to macrophage recruitment and adipose tissue inflammation in obesity. Nature Medicine.

[bib158] Nogueira LM, Lavigne JA, Chandramouli GV, Lui H, Barrett JC, Hursting SD (2012). Dose-dependent effects of calorie restriction on gene expression, metabolism, and tumor progression are partially mediated by insulin-like growth factor-1. Cancer Medicine.

[bib159] Norian LA, Rodriguez PC, O'Mara LA, Zabaleta J, Ochoa AC, Cella M, Allen PM (2009). Tumor-Infiltrating Regulatory Dendritic Cells Inhibit CD8+ T Cell Function via L-Arginine Metabolism. Cancer Research.

[bib160] O'Flanagan CH, Smith LA, McDonell SB, Hursting SD (2017). When less may be more: calorie restriction and response to Cancer therapy. BMC Medicine.

[bib161] O'Neill LA, Hardie DG (2013). Metabolism of inflammation limited by AMPK and pseudo-starvation. Nature.

[bib162] O'Sullivan D, Sanin DE, Pearce EJ, Pearce EL (2019). Metabolic interventions in the immune response to Cancer. Nature Reviews Immunology.

[bib163] Oh J, Magnuson A, Benoist C, Pittet MJ, Weissleder R (2018). Age-related tumor growth in mice is related to integrin α 4 in CD8+ T cells. JCI Insight.

[bib164] Orillion A, Damayanti NP, Shen L, Adelaiye-Ogala R, Affronti H, Elbanna M, Chintala S, Ciesielski M, Fontana L, Kao C, Elzey BD, Ratliff TL, Nelson DE, Smiraglia D, Abrams SI, Pili R (2018). Dietary protein restriction reprograms Tumor-Associated macrophages and enhances immunotherapy. Clinical Cancer Research.

[bib165] Padrón Á, Hurez V, Gupta HB, Clark CA, Pandeswara SL, Yuan B, Svatek RS, Turk MJ, Drerup JM, Li R, Curiel TJ (2018). Age effects of distinct immune checkpoint blockade treatments in a mouse melanoma model. Experimental Gerontology.

[bib166] Painter SD, Ovsyannikova IG, Poland GA (2015). The weight of obesity on the human immune response to vaccination. Vaccine.

[bib167] Park YJ, Kuen DS, Chung Y (2018). Future prospects of immune checkpoint blockade in Cancer: from response prediction to overcoming resistance. Experimental & Molecular Medicine.

[bib168] Pawelec G (2019). Does patient age influence anti-cancer immunity?. Seminars in Immunopathology.

[bib169] Pearce EL, Walsh MC, Cejas PJ, Harms GM, Shen H, Wang LS, Jones RG, Choi Y (2009). Enhancing CD8 T-cell memory by modulating fatty acid metabolism. Nature.

[bib170] Peng M, Yin N, Chhangawala S, Xu K, Leslie CS, Li MO (2016). Aerobic glycolysis promotes T helper 1 cell differentiation through an epigenetic mechanism. Science.

[bib171] Pennock ND, White JT, Cross EW, Cheney EE, Tamburini BA, Kedl RM (2013). T cell responses: naïve to memory and everything in between. Advances in Physiology Education.

[bib172] Pietrocola F, Pol J, Vacchelli E, Rao S, Enot DP, Baracco EE, Levesque S, Castoldi F, Jacquelot N, Yamazaki T, Senovilla L, Marino G, Aranda F, Durand S, Sica V, Chery A, Lachkar S, Sigl V, Bloy N, Buque A, Falzoni S, Ryffel B, Apetoh L, Di Virgilio F, Madeo F, Maiuri MC, Zitvogel L, Levine B, Penninger JM, Kroemer G (2016). Caloric restriction mimetics enhance anticancer immunosurveillance. Cancer Cell.

[bib173] Pollizzi KN, Patel CH, Sun IH, Oh MH, Waickman AT, Wen J, Delgoffe GM, Powell JD (2015). mTORC1 and mTORC2 selectively regulate CD8⁺ T cell differentiation. Journal of Clinical Investigation.

[bib174] Pollizzi KN, Sun I-H, Patel CH, Lo Y-C, Oh M-H, Waickman AT, Tam AJ, Blosser RL, Wen J, Delgoffe GM, Powell JD (2016). Asymmetric inheritance of mTORC1 kinase activity during division dictates CD8+ T cell differentiation. Nature Immunology.

[bib175] Pollizzi KN, Powell JD (2014). Integrating canonical and metabolic signalling programmes in the regulation of T cell responses. Nature Reviews Immunology.

[bib176] Poropatich K, Fontanarosa J, Samant S, Sosman JA, Zhang B (2017). Cancer immunotherapies: are they as effective in the elderly?. Drugs & Aging.

[bib177] Provinciali M, Argentati K, Tibaldi A (2000). Efficacy of Cancer gene therapy in aging: adenocarcinoma cells engineered to release IL-2 are rejected but do not induce tumor specific immune memory in old mice. Gene Therapy.

[bib178] Provinciali M, Smorlesi A, Donnini A, Bartozzi B, Amici A (2003). Low effectiveness of DNA vaccination against HER-2/neu in ageing. Vaccine.

[bib179] Quail DF, Olson OC, Bhardwaj P, Walsh LA, Akkari L, Quick ML, Chen I-C, Wendel N, Ben-Chetrit N, Walker J, Holt PR, Dannenberg AJ, Joyce JA (2017). Obesity alters the lung myeloid cell landscape to enhance breast Cancer metastasis through IL5 and GM-CSF. Nature Cell Biology.

[bib180] Quinn KM, Fox A, Harland KL, Russ BE, Li J, Nguyen THO, Loh L, Olshanksy M, Naeem H, Tsyganov K, Wiede F, Webster R, Blyth C, Sng XYX, Tiganis T, Powell D, Doherty PC, Turner SJ, Kedzierska K, La Gruta NL (2018). Age-Related decline in primary CD8^+^T Cell Responses Is Associated with the Development of Senescence in Virtual Memory CD8^+^ T Cells. Cell Reports.

[bib181] Quinn KM, Palchaudhuri R, Palmer CS, La Gruta NL (2019). The clock is ticking: the impact of ageing on T cell metabolism. Clinical & Translational Immunology.

[bib182] Quinn KM, Hussain T, Kraus F, Formosa LE, Lam WK, Dagley MJ, Saunders EC, Assmus LM, Wynne-Jones E, Loh L, van de Sandt CE, Cooper L, Good-Jacobson KL, Kedzierska K, Mackay LK, McConville MJ, Ramm G, Ryan MT, La Gruta NL (2020). Metabolic characteristics of CD8^+^T cell subsets in young and aged individuals are not predictive of functionality. Nature Communications.

[bib183] Rao E, Zhang Y, Zhu G, Hao J, Persson XM, Egilmez NK, Suttles J, Li B (2015). Deficiency of AMPK in CD8+ T cells suppresses their anti-tumor function by inducing protein phosphatase-mediated cell death. Oncotarget.

[bib184] Rebeles J, Green WD, Alwarawrah Y, Nichols AG, Eisner W, Danzaki K, MacIver NJ, Beck MA (2019). Obesity-Induced changes in T-Cell metabolism are associated with impaired memory T-Cell response to influenza and are not reversed with weight loss. The Journal of Infectious Diseases.

[bib185] Recino A, Barkan K, Wong FS, Ladds G, Cooke A, Wallberg M (2017). Hyperglycaemia does not affect antigen-specific activation and cytolytic killing by CD8^+^ T cells *in vivo*. Bioscience Reports.

[bib186] Renkema KR, Li G, Wu A, Smithey MJ, Nikolich-Žugich J (2014). Two separate defects affecting true naive or virtual memory T cell precursors combine to reduce naive T cell responses with aging. The Journal of Immunology.

[bib187] Rivadeneira DB, DePeaux K, Wang Y, Kulkarni A, Tabib T, Menk AV, Sampath P, Lafyatis R, Ferris RL, Sarkar SN, Thorne SH, Delgoffe GM (2019). Oncolytic viruses engineered to enforce leptin expression reprogram Tumor-Infiltrating T cell metabolism and promote tumor clearance. Immunity.

[bib188] Rodriguez PC, Quiceno DG, Zabaleta J, Ortiz B, Zea AH, Piazuelo MB, Delgado A, Correa P, Brayer J, Sotomayor EM, Antonia S, Ochoa JB, Ochoa AC (2004). Arginase I production in the tumor microenvironment by mature myeloid cells inhibits T-cell receptor expression and antigen-specific T-cell responses. Cancer Research.

[bib189] Ron-Harel N, Sharpe AH, Haigis MC (2015). Mitochondrial metabolism in T cell activation and senescence: a mini-review. Gerontology.

[bib190] Ron-Harel N, Santos D, Ghergurovich JM, Sage PT, Reddy A, Lovitch SB, Dephoure N, Satterstrom FK, Sheffer M, Spinelli JB, Gygi S, Rabinowitz JD, Sharpe AH, Haigis MC (2016). Mitochondrial biogenesis and proteome remodeling promote One-Carbon metabolism for T cell activation. Cell Metabolism.

[bib191] Ron-Harel N, Notarangelo G, Ghergurovich JM, Paulo JA, Sage PT, Santos D, Satterstrom FK, Gygi SP, Rabinowitz JD, Sharpe AH, Haigis MC (2018). Defective respiration and one-carbon metabolism contribute to impaired naïve T cell activation in aged mice. PNAS.

[bib192] Rosenberg SA, Restifo NP (2015). Adoptive cell transfer as personalized immunotherapy for human Cancer. Science.

[bib193] Ross MH, Bras G (1965). Tumor Incidence Patterns and Nutrition in the Rat. The Journal of Nutrition.

[bib194] Rossjohn J, Gras S, Miles JJ, Turner SJ, Godfrey DI, McCluskey J (2015). T cell antigen receptor recognition of antigen-presenting molecules. Annual Review of Immunology.

[bib195] Rous P (1914). The influence of diet on transplanted and spontaneous mouse tumors. Journal of Experimental Medicine.

[bib196] Rubio-Patiño C, Bossowski JP, De Donatis GM, Mondragón L, Villa E, Aira LE, Chiche J, Mhaidly R, Lebeaupin C, Marchetti S, Voutetakis K, Chatziioannou A, Castelli FA, Lamourette P, Chu-Van E, Fenaille F, Avril T, Passeron T, Patterson JB, Verhoeyen E, Bailly-Maitre B, Chevet E, Ricci JE (2018). Low-Protein diet induces IRE1α-Dependent anticancer immunosurveillance. Cell Metabolism.

[bib197] Ruby CE, Weinberg AD (2009). OX40-enhanced tumor rejection and effector T cell differentiation decreases with age. The Journal of Immunology.

[bib198] Ruhland MK, Loza AJ, Capietto AH, Luo X, Knolhoff BL, Flanagan KC, Belt BA, Alspach E, Leahy K, Luo J, Schaffer A, Edwards JR, Longmore G, Faccio R, DeNardo DG, Stewart SA (2016). Stromal senescence establishes an immunosuppressive microenvironment that drives tumorigenesis. Nature Communications.

[bib199] Rytter MJ, Kolte L, Briend A, Friis H, Christensen VB (2014). The immune system in children with malnutrition--a systematic review. PLOS ONE.

[bib200] Salminen A, Kauppinen A, Kaarniranta K (2019). AMPK activation inhibits the functions of myeloid-derived suppressor cells (MDSC): impact on Cancer and aging. Journal of Molecular Medicine.

[bib201] Saucillo DC, Gerriets VA, Sheng J, Rathmell JC, Maciver NJ (2014). Leptin metabolically licenses T cells for activation to link nutrition and immunity. The Journal of Immunology.

[bib202] Sceneay J, Goreczny GJ, Wilson K, Morrow S, DeCristo MJ, Ubellacker JM, Qin Y, Laszewski T, Stover DG, Barrera V, Hutchinson JN, Freedman RA, Mittendorf EA, McAllister SS (2019). Interferon signaling is diminished with age and is associated with immune checkpoint blockade efficacy in Triple-Negative breast Cancer. Cancer Discovery.

[bib203] Scharping NE, Menk AV, Moreci RS, Whetstone RD, Dadey RE, Watkins SC, Ferris RL, Delgoffe GM (2016). The tumor microenvironment represses T cell mitochondrial biogenesis to drive intratumoral T cell metabolic insufficiency and dysfunction. Immunity.

[bib204] Scharping NE, Menk AV, Whetstone RD, Zeng X, Delgoffe GM (2017). Efficacy of PD-1 blockade is potentiated by Metformin-Induced reduction of tumor hypoxia. Cancer Immunology Research.

[bib205] Sekido K, Tomihara K, Tachinami H, Heshiki W, Sakurai K, Moniruzzaman R, Imaue S, Fujiwara K, Noguchi M (2019). Alterations in composition of immune cells and impairment of anti-tumor immune response in aged oral cancer-bearing mice. Oral Oncology.

[bib206] Shankaran V, Ikeda H, Bruce AT, White JM, Swanson PE, Old LJ, Schreiber RD (2001). IFNgamma and lymphocytes prevent primary tumour development and shape tumour immunogenicity. Nature.

[bib207] Sharma S, Dominguez AL, Lustgarten J (2006). High accumulation of T regulatory cells prevents the activation of immune responses in aged animals. The Journal of Immunology.

[bib208] Sheridan PA, Paich HA, Handy J, Karlsson EA, Hudgens MG, Sammon AB, Holland LA, Weir S, Noah TL, Beck MA (2012). Obesity is associated with impaired immune response to influenza vaccination in humans. International Journal of Obesity.

[bib209] Shirakawa K, Yan X, Shinmura K, Endo J, Kataoka M, Katsumata Y, Yamamoto T, Anzai A, Isobe S, Yoshida N, Itoh H, Manabe I, Sekai M, Hamazaki Y, Fukuda K, Minato N, Sano M (2016). Obesity accelerates T cell senescence in murine visceral adipose tissue. Journal of Clinical Investigation.

[bib210] Siegel RL, Miller KD, Jemal A (2019). Cancer statistics, 2019. CA: A Cancer Journal for Clinicians.

[bib211] Smith-Garvin JE, Koretzky GA, Jordan MS (2009). T cell activation. Annual Review of Immunology.

[bib212] Spielmann G, Johnston CA, O'Connor DP, Foreyt JP, Simpson RJ (2014). Excess body mass is associated with T cell differentiation indicative of immune ageing in children. Clinical & Experimental Immunology.

[bib213] Spranger S, Bao R, Gajewski TF (2015). Melanoma-intrinsic β-catenin signalling prevents anti-tumour immunity. Nature.

[bib214] Springer NL, Iyengar NM, Bareja R, Verma A, Jochelson MS, Giri DD, Zhou XK, Elemento O, Dannenberg AJ, Fischbach C (2019). Obesity-Associated extracellular matrix remodeling promotes a macrophage phenotype similar to Tumor-Associated macrophages. The American Journal of Pathology.

[bib215] Sullivan MR, Danai LV, Lewis CA, Chan SH, Gui DY, Kunchok T, Dennstedt EA, Vander Heiden MG, Muir A (2019). Quantification of microenvironmental metabolites in murine cancers reveals determinants of tumor nutrient availability. eLife.

[bib216] Taylor AK, Cao W, Vora KP, De La Cruz J, Shieh WJ, Zaki SR, Katz JM, Sambhara S, Gangappa S (2013). Protein energy malnutrition decreases immunity and increases susceptibility to influenza infection in mice. The Journal of Infectious Diseases.

[bib217] Tilstra JS, Robinson AR, Wang J, Gregg SQ, Clauson CL, Reay DP, Nasto LA, St Croix CM, Usas A, Vo N, Huard J, Clemens PR, Stolz DB, Guttridge DC, Watkins SC, Garinis GA, Wang Y, Niedernhofer LJ, Robbins PD (2012). NF-κB inhibition delays DNA damage-induced senescence and aging in mice. Journal of Clinical Investigation.

[bib218] Tivol EA, Borriello F, Schweitzer AN, Lynch WP, Bluestone JA, Sharpe AH (1995). Loss of CTLA-4 leads to massive lymphoproliferation and fatal multiorgan tissue destruction, revealing a critical negative regulatory role of CTLA-4. Immunity.

[bib219] Tsai S, Clemente-Casares X, Zhou AC, Lei H, Ahn JJ, Chan YT, Choi O, Luck H, Woo M, Dunn SE, Engleman EG, Watts TH, Winer S, Winer DA (2018). Insulin Receptor-Mediated stimulation boosts T cell immunity during inflammation and infection. Cell Metabolism.

[bib220] Turbitt WJ, Collins SD, Meng H, Rogers CJ (2019). Increased Adiposity Enhances the Accumulation of MDSCs in the Tumor Microenvironment and Adipose Tissue of Pancreatic Tumor-Bearing Mice and in Immune Organs of Tumor-Free Hosts. Nutrients.

[bib221] Turbitt WJ, Buchta Rosean C, Weber KS, Norian LA (2020). Obesity and CD8 T cell metabolism: implications for anti-tumor immunity and Cancer immunotherapy outcomes. Immunological Reviews.

[bib222] Uyttenhove C, Pilotte L, Théate I, Stroobant V, Colau D, Parmentier N, Boon T, Van den Eynde BJ (2003). Evidence for a tumoral immune resistance mechanism based on tryptophan degradation by indoleamine 2,3-dioxygenase. Nature Medicine.

[bib223] Vabret N, Britton GJ, Gruber C, Hegde S, Kim J, Kuksin M, Levantovsky R, Malle L, Moreira A, Park MD, Pia L, Risson E, Saffern M, Salomé B, Esai Selvan M, Spindler MP, Tan J, van der Heide V, Gregory JK, Alexandropoulos K, Bhardwaj N, Brown BD, Greenbaum B, Gümüş ZH, Homann D, Horowitz A, Kamphorst AO, Curotto de Lafaille MA, Mehandru S, Merad M, Samstein RM, Sinai Immunology Review Project (2020). Immunology of COVID-19: current state of the science. Immunity.

[bib224] van der Vliet HJJ, Nieuwenhuis EE (2007). IPEX as a result of mutations in FOXP3. Clinical and Developmental Immunology.

[bib225] van der Windt GJ, Everts B, Chang CH, Curtis JD, Freitas TC, Amiel E, Pearce EJ, Pearce EL (2012). Mitochondrial respiratory capacity is a critical regulator of CD8+ T cell memory development. Immunity.

[bib226] Verbist KC, Guy CS, Milasta S, Liedmann S, Kamiński MM, Wang R, Green DR (2016). Metabolic maintenance of cell asymmetry following division in activated T lymphocytes. Nature.

[bib227] Waldman AD, Fritz JM, Lenardo MJ (2020). A guide to Cancer immunotherapy: from T cell basic science to clinical practice. Nature Reviews Immunology.

[bib228] Wanders D, Hobson K, Ji X (2020). Methionine restriction and Cancer biology. Nutrients.

[bib229] Wang R, Dillon CP, Shi LZ, Milasta S, Carter R, Finkelstein D, McCormick LL, Fitzgerald P, Chi H, Munger J, Green DR (2011). The transcription factor myc controls metabolic reprogramming upon T lymphocyte activation. Immunity.

[bib230] Wang Z, Aguilar EG, Luna JI, Dunai C, Khuat LT, Le CT, Mirsoian A, Minnar CM, Stoffel KM, Sturgill IR, Grossenbacher SK, Withers SS, Rebhun RB, Hartigan-O'Connor DJ, Méndez-Lagares G, Tarantal AF, Isseroff RR, Griffith TS, Schalper KA, Merleev A, Saha A, Maverakis E, Kelly K, Aljumaily R, Ibrahimi S, Mukherjee S, Machiorlatti M, Vesely SK, Longo DL, Blazar BR, Canter RJ, Murphy WJ, Monjazeb AM (2019). Paradoxical effects of obesity on T cell function during tumor progression and PD-1 checkpoint blockade. Nature Medicine.

[bib231] Wang H, Franco F, Tsui YC, Xie X, Trefny MP, Zappasodi R, Mohmood SR, Fernández-García J, Tsai CH, Schulze I, Picard F, Meylan E, Silverstein R, Goldberg I, Fendt SM, Wolchok JD, Merghoub T, Jandus C, Zippelius A, Ho PC (2020). CD36-mediated metabolic adaptation supports regulatory T cell survival and function in tumors. Nature Immunology.

[bib232] Weindruch R, Walford RL (1982). Dietary restriction in mice beginning at 1 year of age: effect on life-span and spontaneous Cancer incidence. Science.

[bib233] Weitman ES, Aschen SZ, Farias-Eisner G, Albano N, Cuzzone DA, Ghanta S, Zampell JC, Thorek D, Mehrara BJ (2013). Obesity impairs lymphatic fluid transport and dendritic cell migration to lymph nodes. PLOS ONE.

[bib234] Winer S, Chan Y, Paltser G, Truong D, Tsui H, Bahrami J, Dorfman R, Wang Y, Zielenski J, Mastronardi F, Maezawa Y, Drucker DJ, Engleman E, Winer D, Dosch HM (2009). Normalization of obesity-associated insulin resistance through immunotherapy. Nature Medicine.

[bib235] Wu Z, McGoogan JM (2020). Characteristics of and important lessons from the coronavirus disease 2019 (COVID-19) Outbreak in China. Jama.

[bib236] Xu H, Cao D, He A, Ge W (2019). The prognostic role of obesity is independent of sex in Cancer patients treated with immune checkpoint inhibitors: a pooled analysis of 4090 Cancer patients. International Immunopharmacology.

[bib237] Yang H, Youm YH, Dixit VD (2009a). Inhibition of thymic adipogenesis by caloric restriction is coupled with reduction in age-related thymic involution. The Journal of Immunology.

[bib238] Yang H, Youm YH, Vandanmagsar B, Rood J, Kumar KG, Butler AA, Dixit VD (2009b). Obesity accelerates thymic aging. Blood.

[bib239] Yang W, Bai Y, Xiong Y, Zhang J, Chen S, Zheng X, Meng X, Li L, Wang J, Xu C, Yan C, Wang L, Chang CC, Chang TY, Zhang T, Zhou P, Song BL, Liu W, Sun SC, Liu X, Li BL, Xu C (2016). Potentiating the antitumour response of CD8(+) T cells by modulating cholesterol metabolism. Nature.

[bib240] Zhang Y, Kurupati R, Liu L, Zhou XY, Zhang G, Hudaihed A, Filisio F, Giles-Davis W, Xu X, Karakousis GC, Schuchter LM, Xu W, Amaravadi R, Xiao M, Sadek N, Krepler C, Herlyn M, Freeman GJ, Rabinowitz JD, Ertl HCJ (2017). Enhancing CD8^+^T cell fatty acid catabolism within a Metabolically Challenging Tumor Microenvironment Increases the Efficacy of Melanoma Immunotherapy. Cancer Cell.

[bib241] Zhang C, Yue C, Herrmann A, Song J, Egelston C, Wang T, Zhang Z, Li W, Lee H, Aftabizadeh M, Li YJ, Lee PP, Forman S, Somlo G, Chu P, Kruper L, Mortimer J, Hoon DSB, Huang W, Priceman S, Yu H (2020). STAT3 Activation-Induced fatty acid oxidation in CD8^+^ T Effector Cells Is Critical for Obesity-Promoted Breast Tumor Growth. Cell Metabolism.

[bib242] Zhou J, Tang Z, Gao S, Li C, Feng Y, Zhou X (2020). Tumor-Associated macrophages: recent insights and therapies. Frontiers in Oncology.

[bib243] Zinkernagel RM, Doherty PC (1974). Restriction of in vitro T cell-mediated cytotoxicity in lymphocytic choriomeningitis within a syngeneic or semiallogeneic system. Nature.

